# Noninvasive measurement of glucose and lactate in thermoregulatory sweat in neonates and adults

**DOI:** 10.1126/sciadv.aec4166

**Published:** 2026-07-15

**Authors:** Xinyue Liu, Sally A. N. Gowers, Batia Gourin, Zoe McClure, Sarah Jane Dado, Theresa Abraham, Jayanta Banerjee, Martyn G. Boutelle

**Affiliations:** ^1^ Department of Bioengineering, Imperial College London, UK.; ^2^ Department of Neonatology, Women and Children’s Division, Imperial College Healthcare Trust, UK.

## Abstract

Premature neonates are particularly vulnerable and therefore require rigorous monitoring of analytes such as glucose and lactate. Traditionally, this is achieved through invasive, intermittent blood sampling, which is painful and may cause potential complications. To address this issue, we have developed a noninvasive perfusable skin sampling patch designed to collect analytes from low-volume thermoregulatory sweat without the need for external sweat induction. This can be combined with microfluidic biosensor analysis technologies to detect skin glucose and lactate levels. We were able to demonstrate the potential of this technology for noninvasive measurement of glucose and lactate in premature neonates. Our results showed that skin glucose levels correlated well with time-matched blood glucose measurements. Lactate skin measurements did not correlate with blood measurements, indicating a more complex relationship. In addition, we successfully measured skin glucose levels in healthy adults, which correlated with blood glucose levels for each individual. In contrast with the neonate measurements, the relationship between blood and skin glucose varies between individual adults. We successfully carried out proof-of-concept experiments to detect glucose in real-time in both neonates and in healthy adults by connecting the sampling patch to a continuous analysis system. These results provide the groundwork for subsequent large-scale clinical trials intended to validate the system reliability and clinical applicability within health care contexts, particularly neonatal intensive care units.

## Teaser

In both premature babies and adults, blood glucose can be estimated from skin glucose measured with a noninvasive patch.

## INTRODUCTION

Approximately 1 in 10 newborn babies are admitted to neonatal intensive care units (NICU) in the US (*1*) and 1 in 7 in the UK (*2*, *3*), and almost all require routine monitoring of key metabolite levels, such as glucose and lactate, in blood to detect a range of critical conditions. Abnormal glucose metabolism is common in preterm babies, especially those who are growth restricted, leading to extreme fluctuations in their blood glucose levels (both hypo- and hyperglycemia). This can lead to severe short- and long-term morbidities such as seizures and brain injury, including cerebral palsy and central blindness, if left undetected (*4*). Similarly, serum lactate monitoring is vital in detecting compromised tissue perfusion in congenital heart defects and sepsis, which can be life-threatening.

Routine measurement of blood glucose and lactate in a clinical setting in neonates is carried out by intermittent heel-prick or arterial sampling. These are time consuming for staff and require invasive and painful procedures for vulnerable babies; the pain associated with heel-prick tests has been found to correlate with adverse neurological development (*5*). In addition, repeated tests can mean that blood transfusions are needed to replenish the blood lost due to the baby’s low blood volume. The intermittent nature of the measurements can lead to undetected dangerous metabolite levels in the interim leading to delayed intervention (*6*).

Continuous measurement of key analytes, particularly glucose, in real time would facilitate early detection of pathophysiological changes such as hypo- and hyperglycemia, reduce the number of blood measurements required, and allow clinicians to intervene at the earliest opportunity, ultimately improving patient outcomes (*6*). Continuous glucose monitors (CGMs) are now routinely used for management of diabetes in adults and have been revolutionary in allowing patients to better manage their condition (*7*, *8*). There is growing interest in applying these sensors to continuous monitoring of neonates in the NICU to facilitate better glycemic control. Preliminary studies have shown that CGMs can be beneficial for preterm babies and can help detect periods of prolonged hypo- and hyperglycemia (*9*, *10*). However, CGMs remain invasive, can only measure one analyte, and, as they were developed for adults, are relatively large for a newborn baby (*11*). A customized, noninvasive approach that can measure multiple analytes continuously in this vulnerable population is required.

Sweat-based sensing in adults has received considerable interest in the literature as it has the potential to provide a noninvasive means to detect physiological and pathophysiological changes in the body (*12*–*15*). Most developments in this field rely on active sweat stimulation by means of exercise (*16*, *17*), chemical stimulation (*18*, *19*), or heat treatment (*20*, *21*). This requirement for active stimulation of sweat substantially limits the routine use of such sensors, particularly in a clinical environment and for certain user groups such as neonates or the elderly. Stimulated sweat has been shown to affect sweat composition (*22*), and iontophoretic stimulation, in particular, has limited use due to concerns over skin irritation (*23*). Devices that allow measurement of analytes in nonstimulated sweat and that do not impede routine care would be highly advantageous for clinical applications. Although some studies have found a correlation between sweat metabolite and blood metabolite levels (*17*, *24*–*28*), this relationship remains controversial (*15*, *29*–*31*).

In recent years, researchers have focused on measuring analytes in natural thermoregulatory sweat as a promising alternative to stimulating active sweating (*24*, *25*, *32*–*39*). Thermoregulatory sweat is continually secreted even at rest at very low flow rates [∼0.1 nl/min per gland (*12*)] to regulate body temperature and provides a promising means of noninvasive analyte measurement. However, the low flow rates of thermoregulatory sweat present a challenge in carrying out continuous measurements in terms of providing enough liquid to cover the sensing area (*31*). Studies have targeted body regions with high sweat gland density, such as the fingertip, to increase sweat volume (*24*, *25*, *32*). An alternative solution to this issue would be miniaturization of the sensing area. Nevertheless, truly continuous measurement of metabolites in thermoregulatory sweat, which is essential for effective management of severe metabolite fluctuations in neonates, remains a challenge.

In this study, we present a noninvasive perfusable sampling device that sits on the skin. This sampling patch includes a semipermeable membrane that is perfused at low flow rates, providing a liquid interface to facilitate molecular exchange between low-volume thermoregulatory sweat and the device. This approach overcomes many of the challenges associated with making continuous measurements in thermoregulatory sweat and offers more flexibility in device placement regardless of sweat gland density. In this work, we demonstrate the promise of this approach for noninvasive neonate monitoring of glucose and lactate in the NICU by collecting samples for offline analysis and in proof-of-concept experiments, carrying out real-time continuous measurements. This approach is particularly useful for neonates because of their clinical need and since active sweat stimulation is not practical for this patient group. In addition, we also demonstrate the potential of this approach beyond neonates, for noninvasive glucose monitoring in healthy adults.

## RESULTS AND DISCUSSION

### Measurement system

**Fig. 1.**
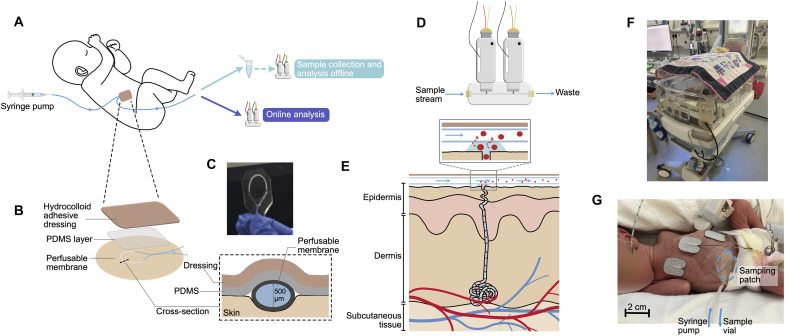
Skin sampling patch and analysis platform for noninvasive monitoring in the NICU. (**A**) Schematic of overall setup in the NICU. The sampling patch was placed on the baby’s abdomen and was perfused with a syringe pump at 0.5 μl/min for offline sample collection and at 0.3 μl/min for online measurements. The outlet of the sampling patch was either connected directly to the analysis system for online analysis or collected into microvials every 20 to 120 min for offline analysis using the same analysis system. (**B**) Schematic of the sampling patch. It consists of a perfusable membrane placed directly on the skin, followed by a layer of PDMS to cover the top of the membrane, and a layer of hydrocolloid adhesive dressing to secure the patch to the skin. The cross-section shows how the membrane is pushed into contact with the skin and is covered by a layer of flexible PDMS and a hydrocolloid dressing. (**C**) Photo of the perfusable membrane on a layer of PDMS. (**D**) 3D-printed microfluidic chip housing a glucose biosensor and lactate biosensor for continuous measurement of sample stream (**E**) Schematic of cross-section of skin with sampling patch on top. Gray box shows zoomed-in section, showing diffusion of molecules from the sweat through the membrane into the sampling patch. In practice, the tensioning of the membrane against the skin means that there is no gap between the membrane and the skin. It is exaggerated here to demonstrate the principle of operation. (**F**) Photo showing neonate in an incubator in the NICU ward. (**G**) Photo showing sampling patch on neonate inside an incubator in the NICU. All schematics were drawn by S.A.N.G.

The measurement system consists of a perfusable sampling patch that can be secured on the skin to sample analytes from thermoregulatory sweat. The outlet was either connected to miniature collection vials for analysis at a later time or to our online analysis system as shown in Fig. 1A. The sampling patch is based around a linear clinical microdialysis probe (66 Linear Microdialysis Catheter, MDialysis, Sweden). This cylindrical probe has a 30-mm polyarylethersulphone membrane and a 20-kDa molecular-weight cutoff. The membrane was threaded through a thin sheet of cured polydimethylsiloxane (PDMS) and arranged into a loop so that the inlet and outlet tubes could be held together to reduce the additional tubes placed around the baby. Uncured PDMS was used to secure the tubing in place and was then cured in the oven at 65°C. The PDMS layer was incorporated into the sampling patch to provide protection for the delicate membrane in case the patch had to be removed during routine clinical care. The sampling patch was secured on the skin using a hydrocolloid adhesive dressing. This was placed on top of the PDMS protective layer so as not to damage the membrane. The various layers of the sampling patch are shown in Fig. 1B, and the cross-section demonstrates how the flexible PDMS layer and the larger hydrocolloid dressing along with the rounded perfusable membrane ensures contact with the skin. A photo of the microdialysis probe on the PDMS sheet is shown in Fig. 1C. The microdialysis probe in the sampling patch was perfused using a portable clinical syringe pump (107 MDialysis, Sweden) with a solution containing ions but without the analytes of interest. Analytes diffuse from the thermoregulatory sweat down their concentration gradient into the perfusate, creating a stream of liquid that we can analyze, as shown in Fig. 1E. This liquid was either collected into miniature sample vials for offline analysis or connected directly to our online analysis system. The online analysis system is described in detail in Materials and Methods but briefly consists of a three-dimensionally (3D)–printed microfluidic chip housing in-house enzyme-based glucose and lactate biosensors, as shown in Fig. 1D. The analyte concentration in the sample will be lower than in thermoregulatory sweat and is a function of perfusion flow rate and membrane characteristics. It could also be affected by sweat rate and the mechanism of transport of analyte from blood to sweat. These factors are discussed in more detail later.

### Neonate measurements

Our first step was to establish if it was possible to measure glucose and lactate in neonatal thermoregulatory sweat using our sampling patch without any sweat stimulation. To do this, neonates in the NICU were recruited for skin monitoring. Overall, 28 babies were monitored with birth gestational ages (GAs) ranging from 23 + 3 to 38 + 2 (week + day), and postnatal ages ranging from 2 to 95 days (explanation of ages used is given in the “Neonate protocol” section of Materials and Methods). Once the parents had been consented, the sampling patch was secured onto either the abdomen or back of the baby and perfused with a sterile ionic solution at 0.5 μl/min. The size of the sampling patch was 3 cm by 2 cm. The patch was left for 15 min to account for the delay between the sampling area and the patch outlet. After this time, collection vials were secured to the patch outlet to collect aliquots of sampled sweat every 20 to 120 min. These samples were frozen at −20°C until analysis.

**Fig. 2.**
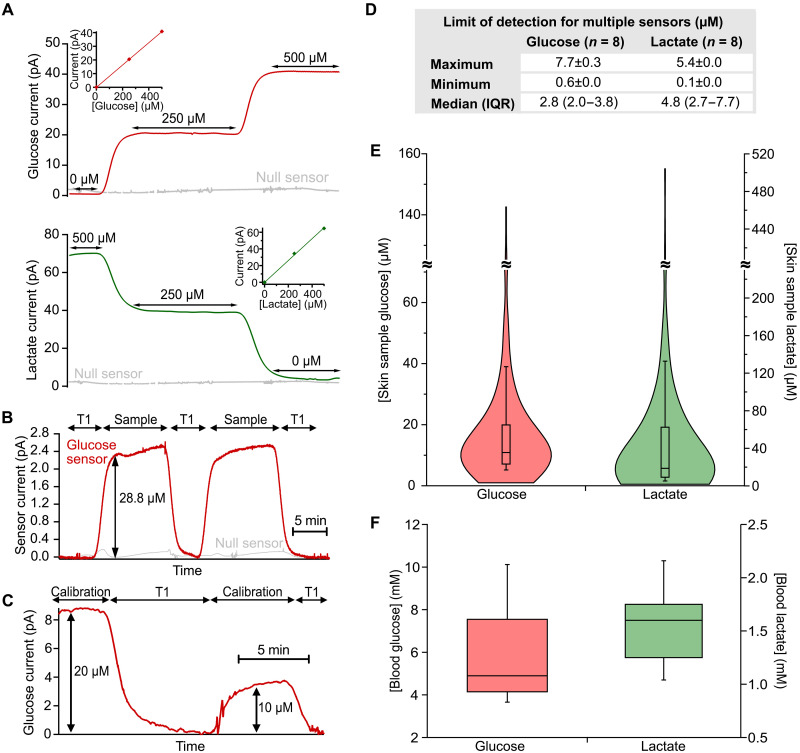
Biosensor calibration, analytical performance, and offline measurements of neonatal skin samples. (**A**) Example traces of glucose (red, top) and lactate (green, bottom) calibrations at 0.5 μl/min. The gray trace shows the response of a null sensor at the same time. Inset shows the corresponding calibration curve relating current to concentration of substrate. (**B**) Example response for a glucose sensor (red) and a null sensor (gray) to two repeat analyses of one neonate skin sample. The microfluidic platform alternates between pushing T1 and the offline sample past the in-house glucose and lactate biosensors (full details in Materials and Methods). All solutions are pushed at 0.5 μl/min. The data were sampled at 10 Hz and smoothed using a Savitsky-Golay 901-point filter. These repeat measurements for one sample are averaged together to improve data reliability. (**C**) Example response of a glucose sensor to a 20 μM glucose change followed by a 10 μM glucose change from baseline. A different, more sensitive, glucose sensor was used giving higher current compared with (B). The sensitivity differs due to differences in the thickness of the polyurethane outer layer. (**D**) Limit of detection values for the eight individually calibrated glucose and lactate biosensors used in the sample analysis. (**E**) Violin plots showing the concentration of glucose (red, *n* = 224 samples) and lactate (green, *n* = 218 samples) in all offline skin samples analyzed from 28 babies. Boxes represent median and IQR, and whiskers represent 10th and 90th percentiles. In total, 19 samples were excluded for glucose and 25 samples are excluded for lactate as their levels were below the limit of detection of the biosensor used for that experiment. (**F**) Box plots (median, IQR, and 10th and 90th percentile whiskers) showing the concentration of glucose (red, *n* = 17 samples) and lactate (green, *n* = 17 samples) for all blood measurements from 15 babies.

Samples were analyzed using our microfluidic platform (fig. S1), comprising programmable syringe pumps and valves from LabSmith for manipulation of the low-volume samples. Our microfluidic flow cell containing a glucose and lactate biosensor was connected to the outlet of the microfluidic sample handling platform. The biosensors were first calibrated using known standards of glucose and lactate. Figure 2A shows an example calibration for both sensors from 0 to 500 μM (glucose shown in red, lactate shown in green). The response of a null sensor to changes in glucose and lactate was negligible, as shown by the gray traces in Fig. 2A. Each sample was then added to a reservoir on the sample handling platform and pushed past the biosensors in the flow cell for analysis. Ten microliters of each sample was analyzed at 0.5 μl/min for 20 min. For larger sample volumes (collected over a longer time period), this analysis was repeated up to three times with T1 perfusion fluid pushed past the sensors in between each repeat. The reservoir was cleaned and dried in between each sample, and T1 solution was pushed through the flow cell in between each sample. Figure 2B shows an example response of a glucose sensor to two repeat analyses of one sample. In this case, two 10-μl aliquots of the sample were analyzed with a 6-μl aliquot of T1 perfusion fluid in between each repeat. The response of a null sensor is also shown in gray and is negligible compared with the size of the glucose sensor response, confirming that the signal at the glucose sensor was not caused by interferents. Figure 2C shows an example of a glucose sensor response to a 20 and 10 μM change from baseline, demonstrating that such changes can be clearly resolved using this setup. It is possible to reliably detect such low levels of these analytes due to the high sensitivity of our sensors; the median limit of detection was 2.8 μM for glucose [*n* = 8 individually calibrated sensors; interquartile range (IQR), 2.0 to 3.8 μM] and 4.8 μM for lactate (*n* = 8 individually calibrated sensors; IQR, 2.7 to 7.7 μM), as summarized in Fig. 2D. Figure S2 shows exemplar levels of glucose and lactate across sequential samples for two babies.

The levels of glucose and lactate measured in the skin samples for all babies are plotted together in the violin plot in Fig. 2E. Glucose could be detected in 92.2% of the samples, and lactate could be detected in 89.7% of the samples; the levels in the remaining samples were below the limit of detection of the particular sensor used and therefore indistinguishable from zero. Based on these measurements, we can conclude that it is possible to detect glucose and lactate in these samples with high confidence (one-sample two-tailed *t* test, *P* < 0.0001). The median concentration in these samples was 10.9 μM (IQR, 7.1 to 19.9 μM) for glucose and 18.6 μM (IQR, 9.1 to 62.4 μM) for lactate. Figure 2F shows the concentration of glucose and lactate in one-off blood measurements made for these babies as part of their routine clinical care; the median blood glucose level was 4.9 mM (IQR, 4.2 to 7.6 mM), and blood lactate level was 1.6 mM (IQR, 1.3 to 1.8 mM). The levels of both glucose and lactate in the skin samples were considerably lower than in the blood samples; this is consistent with glucose being found at lower concentrations in sweat (*28*, *31*) and is likely also to be due partly to dilution into the perfusate. Based on Fig. 2 (E and F), it is clear that there is large variability in the concentration of glucose and lactate across both the skin and blood samples. This could be due to differences in the health of each baby or in variability in sweat function with age, or, as discussed later, this could be due to variability in the timing of the sample compared to the feeding schedule of each baby.

**Fig. 3.**
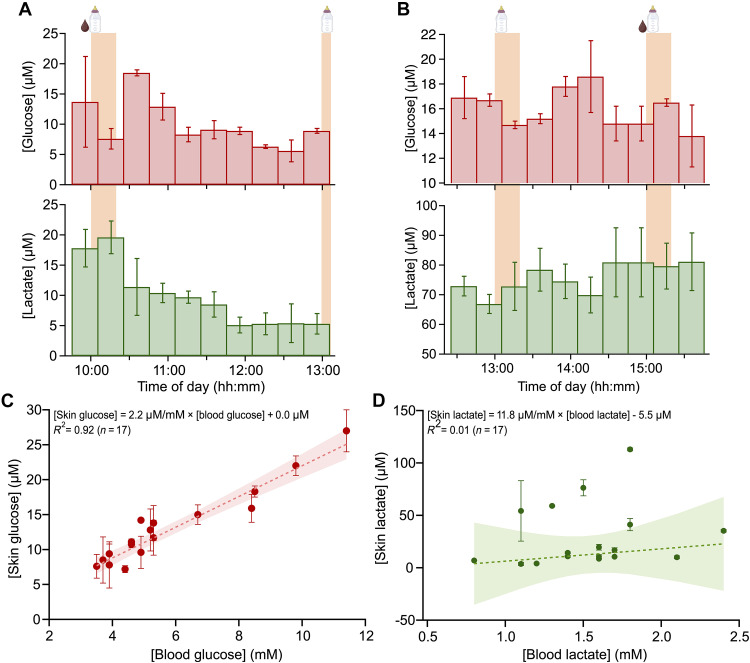
Time-resolved skin glucose and lactate measurements in premature neonates and comparison with blood levels. Examples of glucose (red) and lactate (green) levels in samples collected on the skin of two babies over time. The width of each bar represents the time that sample was collected over (typically 20 min). Bars and error bars represent the mean and standard deviation of the plateau measurement for each sample. Samples have been shifted to account for the delay between the membrane and the sample vial (15 min). Nasogastric feeds, indicated by a milk bottle, typically lasted 20 min. The duration is indicated by the orange bars under the bar graphs. A droplet of blood image indicates when a blood measurement was made. (**A**) In this example, the baby was fed every 3 hours and was 33 + 2 weeks GA and 2 days postnatal age. (**B**) In this example, the baby was 27 + 0 weeks GA and 19 days postnatal age and was fed on a 2-hourly schedule. (**C**) Correlation between skin sample glucose concentration and blood glucose concentration for 17 samples from 15 babies. The points are fitted with a weighted linear regression, *R*^2^ = 0.92 and slope 2.2 ± 0.2 μM/mM. The shaded area represents the 95% confidence interval of the regression line. (**D**) Correlation between skin sample lactate concentration and blood lactate concentration for 17 samples from 15 babies. The points are fitted with a weighted linear regression, *R*^2^ = 0.01 and slope 11.8 ± 0.8 μM/mM. The shaded area represents the 95% confidence interval of the regression line. Blood glucose and lactate are measured routinely as part of standard clinical care using an arterial blood gas analyzer. These measurements are plotted against the sample concentration on the baby’s skin at the same time as the blood sample is taken.

To investigate whether the variability in the sample metabolite levels in Fig. 2E was due to the timing of the sample relative to the feeding schedule of the baby, we reduced the sampling time to 20 min. At a flow rate of 0.5 μl/min, this gives a sample size of 10 μl, which is the minimum volume that can be reliably analyzed using our microfluidic sample handling platform. This shorter collection time provided better time resolution and allowed better correlation with the feeds, which were typically every 2 to 3 hours. Figure 3 (A and B) shows example glucose and lactate levels in sequential samples at 20-min time resolution. These figures clearly show that levels of glucose and lactate fluctuate over time. Glucose in particular seems to change in relation to the feeding schedule; in both cases, glucose increases following a feed and then decreases again until the next feed. The timing of the postfeed glucose peak seems to vary between the two examples shown in Fig. 3 (A and B), presumably due to differences in the babies’ physiology. For each baby, a blood sample was taken at the point indicated by the image of the droplet of blood in Fig. 3 (A and B). For these babies, the glucose concentration for the blood sample corresponding to Fig. 3A was 3.5 mM and for the blood sample corresponding to Fig. 3B was 5.3 mM, which is consistent with the skin sample glucose levels being higher in Fig. 3B than Fig. 3A.

Although lactate levels in these samples do vary over time, the changes do not seem to correlate with the feeding schedule. When these levels are compared with blood lactate levels for both babies, the blood lactate level was the same (1.6 mM); however, the skin sample lactate levels in Fig. 3 (A and B) are quite different, suggesting that any relationship between blood and skin levels is more complicated for lactate. This is supported by other studies in the literature, which suggest that lactate measurements may reflect local lactate production by the sweat gland itself (*15*, *40*, *41*).

As blood levels are likely to vary over time and with feeding, it is important to compare blood and skin samples collected at the same time to establish whether metabolite levels in the skin samples correlate with blood levels; this is possible with the higher resolution sampling. Furthermore, where possible, blood samples were taken immediately before a feed where levels were likely to be more stable. To establish whether a correlation between blood and skin sample glucose exists, the glucose level in the skin sample is plotted against the blood glucose level taken at the same time, as shown in Fig. 3C. For most babies, only one blood sample was taken during the monitoring period, but in two cases, two samples were taken. Figure 3C shows a strong relationship between blood glucose levels and glucose levels in the concomitant skin samples, with a correlation coefficient (*R*^2^) value of 0.92. The slope of the correlation line (2.2 ± 0.2 μM/mM) indicates that the skin sample glucose concentration is 0.22 ± 0.02% of blood glucose levels. This strong correlation is an important result that is unexpected and suggests that noninvasive measurement of glucose in thermoregulatory sweat can provide an alternative means of monitoring blood glucose levels in all neonates including those as young as 23 + 3 weeks gestational (full age details for all babies is given in table S1). As shown in Fig. 3D, lactate levels in the skin samples did not correlate well with concomitant blood lactate levels (*R*^2^ = 0.01), suggesting that the relationship between blood lactate and lactate in the thermoregulatory sweat is less straightforward.

### Adult measurements

The ability to noninvasively measure blood glucose levels via skin sampling of thermoregulatory sweat is particularly important for neonates; however, there are also many clinical applications in adults where a similar approach would be beneficial. Lactate measurement in thermoregulatory sweat seems to be a less promising marker since skin lactate levels did not correlate well with blood lactate levels in neonates. In addition, it is generally understood that lactate concentration in adult sweat is related to local metabolism of the sweat gland rather than the concentration of lactate in the blood (*42*). With this in mind, we sought to establish whether our approach could be used to noninvasively measure blood glucose levels in adults. To test this, we placed the sampling patch on the ventral forearm of healthy adult volunteers at rest. A photo showing an example of the sweat gland density on the ventral forearm of a healthy adult volunteer is shown in fig. S3. In this example, we see a sweat gland density of ∼177 glands/cm^2^, which is broadly similar to the density of 159 glands/cm^2^ described elsewhere (*43*).

**Fig. 4.**
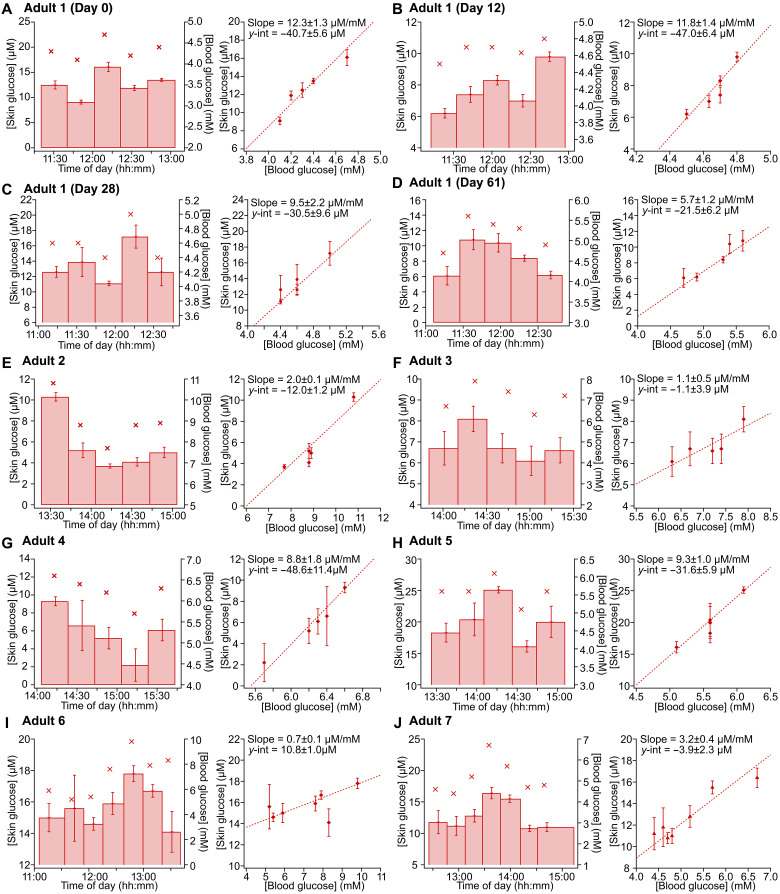
Skin glucose measurements in adults individually correlate with blood glucose. (**A** to **D**) Glucose levels in samples collected on the skin of one adult at rest over four separate occasions (bars) and concomitant blood glucose levels (crosses). Scatter graph shows the correlation between skin sample glucose concentration and blood glucose concentration at each time point. (**E** to **J**) Glucose levels in samples collected on the skin of a further six adults. In each case, each bar represents the time that the sample was collected over. Each sample is 20 min long. Bars and error bars represent the mean and standard deviation of the plateau measurement for each sample. Samples have been shifted to account for the delay between the patch and the sample vial (7 min). Crosses indicate the glucose concentration in one-off finger-prick blood tests, taken roughly midway through each skin sample with a blood glucose analyzer. This is plotted against the sample concentration on the adult’s skin at the same time as the blood sample is taken. The points are fitted with a weighted linear regression for each adult. The regression coefficients ± standard deviation of the slope and *y* intercept (*y*-int) are given. The correlation lines for adult 1 have slopes of 12.3 ± 1.3 μM/mM (*R*^2^ = 0.91) for day 0, 11.8 ± 1.4 μM/mM (*R*^2^ = 0.84) for day 12, 9.5 ± 2.2 μM/mM (*R*^2^ = 0.80) for day 28, and 6.2 ± 1.2 μM/mM (*R*^2^ = 0.82) for day 61. The slopes for adults 2 to 7 are 2.0 ± 0.1 μM/mM (*R*^2^ = 0.82), 1.1 ± 0.5 μM/mM (*R*^2^ = 0.86), 8.8 ± 1.8 μM/mM (*R*^2^ = 0.93), 9.3 ± 1.0 μM/mM (*R*^2^ = 0.87), 0.7 ± 0.1 μM/mM (*R*^2^ = 0.84), and 3.2 ± 0.4 μM/mM (*R*^2^ = 0.84), respectively.

Samples were collected every 20 min, as with the higher–time resolution neonate sampling. The glucose concentration in these samples was later analyzed using our microfluidic sampling handling platform; as with the neonate analysis, the samples were analyzed using in-house glucose and lactate biosensors (full details in Materials and Methods). Blood glucose measurements were also made during each sample, using a finger-prick blood glucose analyzer (True Metrix Go, Trividia). The measured skin sample glucose concentration and concomitant blood glucose concentration for seven adult volunteers are shown in Fig. 4 (A to J), together with the corresponding correlation between blood and skin glucose for each adult (fig. S4 shows the same data but with axis scales the same for comparison). Skin sample glucose levels for adults are a similar order of magnitude to that of the neonate samples, although there is more variability in the relationship between skin and blood levels for adults. For all adults monitored, the skin sample glucose concentration closely tracks the blood glucose concentration, although as with the neonates, the absolute concentration is considerably lower. Within the physiological range, there appears to be a linear relationship between blood and skin glucose levels, although lines do not go through the origin, indicating either a nonlinearity at low blood glucose levels or some consumption of glucose on the skin. Previous studies have predicted a 6-min delay between changes in the blood and sweat (*44*); however, since our samples were collected over 20 min, this delay will not be apparent. These results show that there is a clear correlation between the concentration of glucose in the skin and blood samples for each adult. The slope of this correlation is different for each adult.

**Fig. 5.**
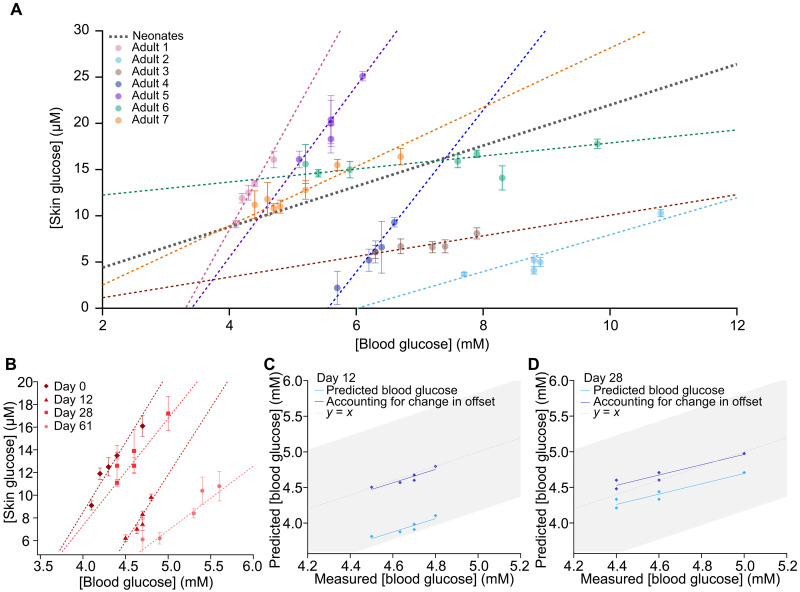
Comparison of skin and blood glucose correlations across adults and over time. (**A**) Overall plot showing correlation between skin sample glucose concentration and blood glucose concentration for all adults compared with neonate correlation line (black dotted line). Markers and error bars represent the mean and standard deviation of the measurement. Points are fitted with a weighted regression line for each adult on a specific day. (**B**) Correlation between skin sample glucose concentration and blood glucose concentration for one adult on four separate occasions, on day 0 (diamond), day 12 (triangle), day 28 (square), and day 61 (circle). Markers and error bars represent the mean and standard deviation of the measurement. Points are fitted with a weighted regression line for each day. (**C**) Correlation between predicted blood glucose levels (light blue) on day 12 against measured blood glucose concentration. Predicted blood glucose concentrations are calculated from skin sample glucose levels using the regression line between skin sample glucose and blood glucose on day 0. Using a one-point blood measurement on day 12, the offset can be accounted for (dark blue). (**D**) Correlation between predicted blood glucose levels (light blue) on day 28 against measured blood glucose concentration. Predicted blood glucose concentrations are calculated from skin sample glucose levels using the regression line between skin sample glucose and blood glucose on day 0. Using a one-point blood measurement on day 28, the offset can be accounted for (dark blue). Markers and error bars represent the mean and standard deviation of each measurement. Points are fitted with a weighted regression line. The gray dotted line represents where predicted blood glucose levels and measured blood glucose levels are equal. The shaded gray area represents the margin of error in the accuracy of the blood glucose analyzer (±0.83 mM for glucose levels <5.55 mM).

Figure 5A shows all the adult correlation slopes together compared with the neonate correlation slope. Unlike the neonate data, when all data points for all adults are considered together (fig. S5), there is no clear correlation between glucose in blood and thermoregulatory sweat. At first sight, it is not clear why the relationship between blood glucose and thermoregulatory sweat glucose is more consistent between babies than between adults, although we know that the skin is more similar between babies, as it is not yet fully developed and babies are in a tightly controlled environment in the NICU, which could partly explain these differences. One possible explanation for the difference in correlation slope between adults could be that different adults have different sweat gland density on their forearms. We carried out a preliminary investigation using bromophenol blue dye (as described in Materials and Methods) to visualize the sweat gland density on three of the adults tested in Fig. 4. The results are shown in table S2. The sweat gland density on the ventral forearms of adults 1 and 4 is considerably higher than adult 6, which is consistent with the steeper correlation slopes for adults 1 and 4 compared with adult 6. This suggests that there may be some effect of sweat gland density. However, the difference in sweat gland density between adults 1 and 4 does not explain the difference in correlation slope for these two adults, suggesting that the relationship is not simple. Further investigation is required to investigate this effect in more detail. The finding that the correlation slope is different for each adult is consistent with findings in a recent study by Saha *et al.* (*24*). Figure S6 shows box plots of both neonate and adult blood and skin sample glucose levels for comparison. It is clear from the overlap in the box plots that adult and neonate levels of both skin sample and blood glucose are comparable.

To investigate whether the relationship between blood and thermoregulatory sweat glucose levels for a particular adult holds true over time, we repeated the sampling experiment on three more occasions over the next 61 days, as shown in Fig. 5B. In all cases, there is a strong correlation between thermoregulatory sweat and blood glucose levels, although this relationship does change slightly over time. As shown in Fig. 5B, the slope of the correlation for days 0 and 12 are very similar, although the *y* intercept of the correlation line has changed. By day 28, the slope of the correlation changed slightly, although not to the extent of the difference between days 0 and 61, and the *y* intercept also changed again.

It is interesting to investigate whether the sweat measurements could be used to predict the blood values at days 12 and 28 based on the correlation line at day 0. The light-blue markers in Fig. 5 (C and D) show the predicted blood glucose values on days 12 and 28, respectively, calculated using the correlation line at day 0, plotted against the actual blood values. In each case, the gray dotted line represents the line if the predicted and actual blood glucose values were the same, and the gray shaded area represents the allowed margin of accuracy on the blood glucose meter (according to EN ISO 15197:2015). There is a strong correlation between predicted and actual blood glucose values on both days 12 and 28, with a slope of 0.94 ± 0.22 and 0.72 ± 0.14, respectively. In both cases, there is an offset resulting in the predicted values slightly underestimating the blood glucose concentrations. This offset is due to a change in the *y* intercept for the correlation line of sweat to blood glucose levels between the two time periods; however, a one-point blood measurement can be used to account for this change in *y* intercept, assuming the slope remains constant. As shown by the dark-blue markers in Fig. 5 (C and D), using a one-point blood measurement on each day to correct for this change in *y* intercept shifts the predicted blood values for both days 12 and 28 much closer to the actual values. This means that once a personalized correlation line has been established, levels of glucose in thermoregulatory sweat could be used to accurately predict blood glucose concentrations up to 28 days later with the need for only a one-off blood measurement. Figure S7 shows that over longer time periods, this prediction becomes less effective.

### Skin/sampling patch interface

The concentration of analyte in the sample will be lower than that in the actual sweat. The final concentration is affected by several factors, including the membrane size and characteristics, as well as the relative rates of the perfusion flow rate and the local sweat rate. Our aim is for the measurement to be independent of sweat rate as this can vary over time.

**Fig. 6.**
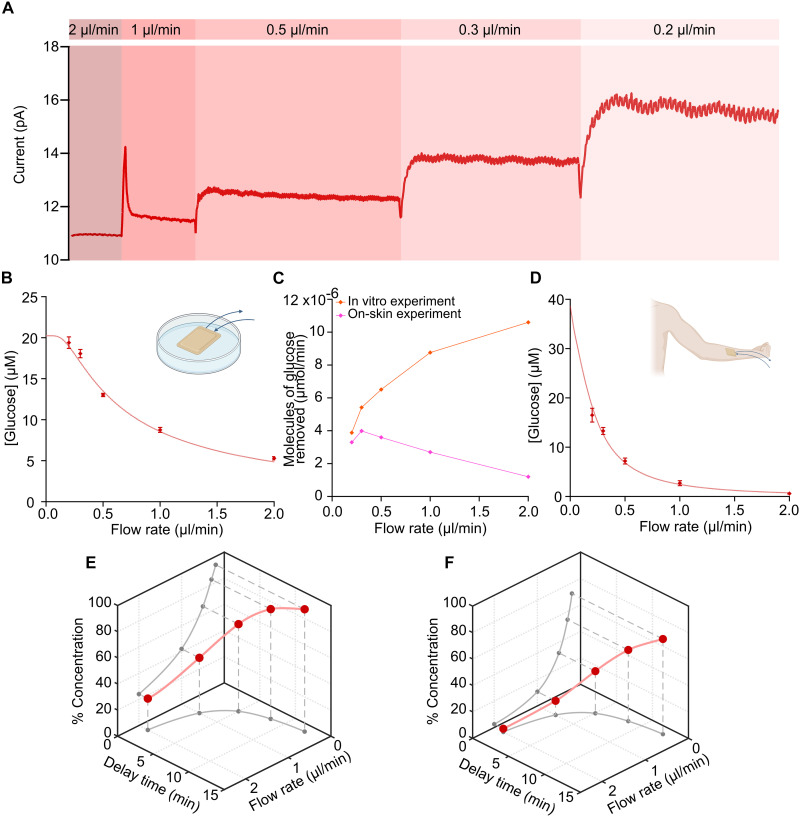
Flow rate dependence of glucose sampling performance in vitro and on the skin. (**A**) Example trace showing how the current at a glucose sensor changes for flow rates from 2 to 0.2 μl/min. The measurements were made in 20 μM glucose. (**B**) Variation in sample glucose concentration with flow rate from 0.2 μl/min to 2 μl/min when sampling patch is placed on a solution of 20 μM glucose in T1 solution. The points are fitted with Eq. 1 (*45*). (**C**) The rate of glucose molecules removed at different flow rates for both the in vitro experiment and the on-skin experiment. (**D**) Variation in sample glucose concentration with flow rate from 0.2 to 2 μl/min when the sampling patch is placed on adult skin at rest. The points are fitted with Eq. 2. In all cases, markers and error bars represent the mean and standard deviation of each measurement once it stabilized. For each measurement the mean current was converted to a glucose concentration using a calibration carried out at the same flow rate. (**E**) 3D plot showing how the percentage concentration of sampled glucose and collection time vary with flow rate for in vitro measurements. Percentage concentration represents the proportion of analyte collected from the sweat, and collection time represents the time needed to collect 10 μl, the minimum volume needed for analysis. (**F**) 3D plot showing how the percentage concentration of sampled glucose and delay time vary with flow rate for on-skin measurements. Percentage concentration represents the proportion of analyte collected from the sweat, and delay time represents the time taken for a concentration change happening at the sampling patch to reach an online analysis system based on an outlet tubing length of 10 cm and inner diameter of 400 μm. Schematics created in BioRender. Liu, X. (2026); https://BioRender.com/zayfdix.

To establish what is happening at the skin/sampling patch interface, and to give insight into the different skin–to–blood glucose relationships for adults compared with babies, we investigated the effect of perfusion flow rate on the resulting sampled glucose concentration. Two experiments were carried out varying the perfusion flow rate, one in vitro on a solution of known glucose concentration and one on the skin of a healthy adult at rest. This was carried out on an adult as opposed to a neonate since it was important that blood glucose levels were stable throughout; as neonates follow a strict feeding schedule with regular feeds, this would not have been possible. The flow rate–dependent recovery of analytes from a bulk concentration through a perfused membrane has been described in detail for microdialysis probes (*45*); this model should describe the in vitro experiment and, by comparison, will help us determine what is happening in the on-skin experiment. In these experiments, the sampling patch was attached to our analysis system for real-time measurement of the glucose concentration in the outflow. The resulting glucose concentration was measured at 0.2, 0.3, 0.5, 1.0, and 2.0 μl/min. Online analysis was used in this case as it meant that it was possible to ensure that the signal had stabilized with each new flow rate before the measurement was taken; this would not have been possible with sample collection. As the glucose sensor is itself flow-rate sensitive, care was taken to carry out a multipoint calibration of the sensors at the corresponding flow rate to convert the measured current into concentration for each flow rate tested. Figure 6A shows an example of how the glucose current varies with flow rate for a glucose sensor in 20 μM glucose.

The results of the in vitro measurements are shown in Fig. 6B. The measurements can be fitted with the equation

$$C_{\text{out}}=C_{\text{ext}}-C_{\text{ext}}e^{\left(-k_{0}A/F\right)}$$ (1)

where *C*_out_ is the concentration of glucose in the sample, *C*_ext_ is the actual concentration of glucose on the skin or in the solution, *k*_0_ is the average mass transfer coefficient, *A* is the area of the probe, and *F* is the flow rate (*45*). This empirical equation describes how the sample concentration of the analyte of interest increases exponentially with decreasing flow rates and can be used to approximate the concentration at which there is no flow or the actual concentration. This relationship assumes that the concentration in the region of the membrane is constant and not changed by the sampling.

As shown in Fig. 6B, when the sampling patch is placed on a solution of known glucose concentration, Eq. 1 does indeed fit the measurements well and estimates the glucose solution as 20.2 ± 0.7 μM, which is very close to the actual concentration (20 μM); it suggests that at 0.5 μl/min, the patch output is 64.4 ± 2.4% of the true concentration. In contrast, the on-skin measurements do not fit the model as well, as shown in fig. S8, suggesting that the interface between the sampling patch and the skin cannot simply be explained by flow rate–dependent diffusion from the sweat into the perfusate. Using the measured concentration at each flow rate, it is possible to calculate the number of molecules removed by the sampling patch in each of the two cases. As shown in Fig. 6C, the in vitro experiment follows the predicted equation, where more molecules are removed as the flow rate increases due to steeper concentration gradients across the membrane (*46*). However, the on-skin experiment does not show the same trend; in this case, less molecules are removed as the flow rate increases. We hypothesize that this is due to a limited supply of glucose as a result of the low resting sweat rates, which cannot keep up with the faster removal rate at higher perfusion flow rates, and hence, less molecules are removed than would be predicted from the in vitro experiment. As a result, at higher flow rates, the relationship between sampled glucose and flow rate appears as though there was a dilution of a fixed flux of molecules into increasing volumes. Hence, the equation has been adapted to account for this, shown in Eq. 2

$$C_{\text{out}}=C_{\text{ext}}\left(\frac{F_{0}}{F_{0}+F}\right)\left[1-e^{\left(-k_{0}A/F\right)}\right]$$ (2)

where *F*_0_ represents the total sweat rate from sweat glands in contact with the sampling membrane. In the case of low perfusion flow rate compared with sweat rate, $\frac{F_{0}}{F_{0}+F}$ tends to 1, giving Eq. 1. In the case of high perfusion flow rate, $\frac{F_{0}}{F_{0}+F}$ tends to $\frac{1}{F}$, giving Eq. 1 multiplied by a flow rate–dependent dilution factor.

As shown in Fig. 6D, with this adapted equation, the on-skin measurements fit the model well. As with the previous example, this can be used to approximate the concentration of glucose on the skin and, hence, the percentage recovered at 0.5 μl/min; based on Fig. 6D, the true skin concentration is 38.6 ± 24.0 μM, and the patch output at 0.5 μl/min is 18.7 ± 11.7%. Since relatively few points were used for the fitting, the error in these approximations is quite large. Nevertheless, the concentration of glucose in thermoregulatory sweat is comparable with that reported in other studies in the literature (*17*, *28*, *44*).

These experiments indicate that the relative magnitude of the glucose removal rate by the membrane and sweat delivery rate to the membrane is very important; at high glucose removal rates, such as a large sensor area or, in our case, higher perfusion flow rates, the concentration of molecules from the sweat region next to the membrane is depleted more quickly than the low thermoregulatory sweat rate can replenish, resulting in concentrations that are dependent on the sweat delivery rate. For a fixed sampling location on an individual adult, this is given by the sweat rate. At the lower glucose removal rates, provided by lower device perfusion rates, concentrations will be independent of sweat delivery rate and, hence, for a fixed sampling location on an individual adult, on sweat rate. This is an important consideration in making measurements in thermoregulatory sweat, as it is critical that results do not depend on sweat rate, which will vary over time.

Eccrine sweat glands form around the third month of gestation; therefore, the number of sweat glands is similar in newborns and adults. However, the smaller skin surface area of babies leads to a higher sweat gland density compared with adults. Although many studies have investigated the sweat gland density in adults (*32*, *43*, *47*, *48*), this has been less well documented for newborn babies. One study found the sweat gland density on the thighs of 7- to 10-day-old babies within 2 weeks of term to be 6.5 times higher than that found in adults (*49*). However, sweat glands are not always fully functional at birth, and the sweating response in newborns is limited compared with adults; it is correlated with both GA and postnatal age (*50*, *51*). As neonates have a higher sweat gland density than adults, they can replenish molecules that have been removed from the sweat more quickly for a given area regardless of sweat rate. Therefore, for babies, we hypothesize that the sweat on the skin would not be depleted at higher sampling flow rates (up to a limit) in the same way it would for adults. This means that the baby measurements would be less dependent on sweat rate and sweat gland density, whereas with adults both these factors would affect the measured sweat concentration. This could explain the higher variability between adults compared with between babies. Lowering the flow rate of our device would remove this risk of variability between adults and put the measurement in a safer zone, meaning that it is less dependent on sweat rate and therefore more suited to making measurements in thermoregulatory sweat. 

Figure 6 clearly shows that the concentration of glucose in the sample greatly increases at lower flow rates. For our initial sample collection study, although lower flow rates would yield higher sample concentrations, it would take longer to collect the minimum sample volume needed for analysis and lead to lower temporal resolution, as shown in Fig. 6E. Therefore, 0.5 μl/min was chosen as a compromise between sample glucose concentration and adequate time resolution. However, with online analysis, the minimum sample volume is no longer a consideration, and hence, lower flow rates can be used to increase the glucose concentration in the analysis stream and to ensure that the concentration is not affected by sweat rate. With online analysis, lower flow rates will still have an impact on the delay between the sampling patch and the analysis system, but using short tubing lengths can minimize this. These considerations are summarized in Fig. 6F. Based on the results in Fig. 6 (C and F), we determined that using a sampling flow rate of 0.3 μl/min for future experiments would provide a good compromise between delay time and ensuring our measurements are in a zone where they are independent of sweat rate.

### Real-time continuous measurements

Given the promise of this sampling method, the next step was to move toward continuous measurements. As described above, for online measurement, it is advantageous to use lower flow rates than used in the offline sample collection, as sample volume is no longer a consideration; a flow rate of 0.3 μl/min was chosen to provide an adequate compromise between glucose concentration and time delay. In reality, the actual flow rate was quite variable due to the portable syringe pump used and was typically slightly lower than the target flow rate, as shown in fig. S9 for different experimental conditions. We combined the sampling patch with a continuous microfluidic analysis system developed previously by our group (*52*, *53*), shown in Fig. 1D, for online glucose measurement. As described above, we predict that the lower perfusion flow rate will result in higher glucose concentrations in the analysis stream.

**Fig. 7.**
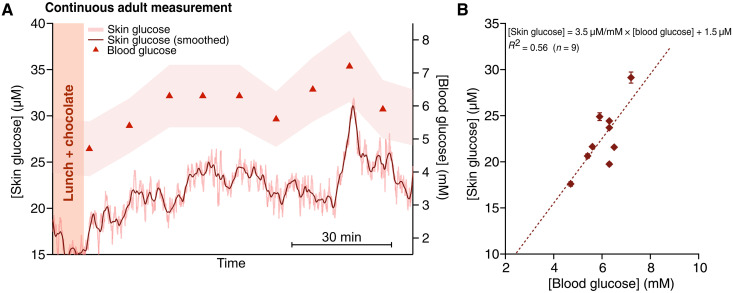
Real-time online measurement of skin glucose in an adult. (**A**) Real-time glucose concentration on the ventral forearm of a healthy adult over time. The light red trace shows raw data sampled at 10 Hz, and the darker red overlaid trace shows data smoothed with a Savitsky-Golay 2001-point filter. Triangles represent discrete blood glucose measurements carried out using a finger-prick blood glucose meter. The shaded area represents the margin of error in the accuracy of the blood glucose analyzer (±0.83 mM for glucose levels <5.55 mM, and ±15% of the reading for above or equal to 5.55 mM). Skin measurements have been shifted to account for the delay between the patch and the analysis system (8 min). Food was consumed at the beginning of the monitoring and is indicated by the orange shaded rectangle labeled lunch and chocolate. (**B**) Scatter graph showing the correlation between skin glucose concentration (averaged over a 5-min window) and blood glucose concentration at each time point. Markers and error bars represent the mean and standard deviation of the measurement. Points are fitted with a weighted regression line. The correlation line has a slope of 3.5 ± 0.5 μM/mM (*R*^2^ = 0.56).

To demonstrate the potential of this method for continuous glucose measurement, we first monitored a healthy adult at rest and measured the skin glucose level continuously after eating food. The sampling patch was positioned on the ventral forearm, and the outflow was connected to a 3D-printed microfluidic chip housing a glucose biosensor connected to a wireless potentiostat. Frequent finger-prick blood glucose measurements were also made to compare with the skin measurements. Figure 7A shows the skin glucose level over time along with the discrete blood glucose measurements. The results show an initial increase in both blood and skin glucose levels after eating, which peaks around half an hour after food intake. There is then a second peak around 45 min after the first peak. We hypothesize that these two peaks are due to the combination of fast- and slow-release carbohydrates that were consumed. Regardless, it is promising that the skin measurements track the changes measured in the blood. Figure 7B summarizes the correlation between blood and skin glucose measurements (averaged over a 5-min window) over the monitoring period. As seen in the offline analysis, there was a linear correlation between skin glucose and blood glucose levels. Although there is a clear correlation, the *R*^2^ value is lower than for the offline sampling measurements; we hypothesize that this is because we have not accounted for any delay between blood and sweat measurements, and such discrepancies would be more apparent in the higher time-resolution data. Work is ongoing to quantify this delay. The continuous skin measurements can be averaged into 20-min blocks to mimic the offline sampling carried out initially. As shown in fig. S10, these averaged levels do still track blood levels, but the dynamic information regarding glucose changes is lost.

**Fig. 8.**
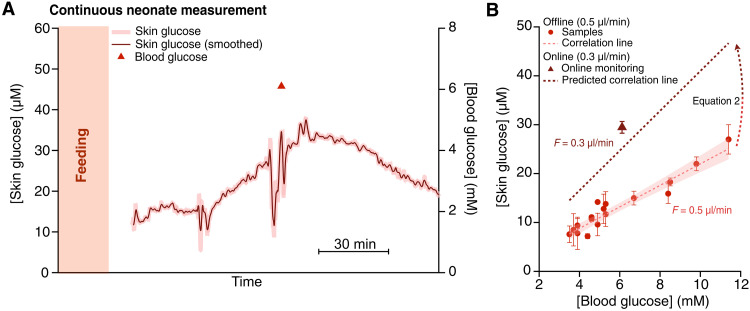
Real-time online measurement of skin glucose in a premature neonate. (**A**) Real-time measurement of skin glucose concentration on the back of a premature infant with GA of 29 + 1 weeks. Light red shows raw data sampled at 10 points/s, and darker red overlaid trace shows data smoothed with a Savitsky-Golay 2001-point filter. The triangle represents a discrete blood glucose measurement carried out using an arterial blood gas analyzer. Skin measurements have been shifted to account for the delay between the patch and the analysis system (30 min). The infant was fed every 2 hours. (**B**) Previously established correlation between skin glucose and blood glucose measurements for offline analysis at 0.5 μl/min (red) and predicted correlation at flow rate of 0.3 μl/min (burgundy). The predicted correlation was calculated using the established relationship between concentration and flow rate described by Eq. 2. The equation of the predicted correlation line is [sweat glucose] = 4.0 μM/mM × [blood glucose] + 0.0 μM. A time-matched measurement of blood and skin glucose levels recorded at the flow rate of 0.3 μl/min is represented by a burgundy triangle.

Having demonstrated the potential of this method for continuous noninvasive glucose measurement in healthy adults, our next aim was to show proof of concept that this method could be used to noninvasively measure glucose in neonates in the NICU. The logistics of monitoring a baby in an enclosed incubator while still allowing for normal clinical care meant that longer outlet tubing was required than was needed for stationary adults. The longer outlet tubing resulted in higher back pressure, and in some cases, this caused either the sampling patch or the analysis system to leak, resulting in loss of data. This back pressure would be significantly improved by reengineering the analysis system to create a miniaturized wearable integrated patch that does not require long extension tubing. Nevertheless, we were able to successfully measure skin glucose continuously for limited periods of time in babies in the NICU. Figure 8A shows an example of continuous skin glucose measurements in a neonate over a 2-hour window. The baby was given a feed just beforehand as indicated by the orange box, and another toward the end of the monitoring. A routine blood glucose measurement is also plotted for comparison. In this example, the skin glucose level increased around 40 min after the end of the feed and was beginning to decrease before another feed was given. As expected, the skin glucose levels are higher than seen in the offline analysis due to the lower flow rate used in this case as discussed above. Figure 8B shows the correlation line established in the offline neonate study (red) at 0.5 μl/min along with how we would predict the correlation line would change for the lower flow rate of 0.3 μl/min (burgundy) using Eq. 2. The one-off blood glucose measurement is plotted against the time-matched skin glucose concentration (mean over 5 min) as a burgundy triangle in Fig. 8B; the point lies very close to that predicted for this lower flow rate.

In this work, we have developed a noninvasive sampling patch that can be combined with offline or online analysis methods to measure levels of key analytes such as glucose and lactate in thermoregulatory sweat as an alternative to invasive blood measurements. We have demonstrated the use of this sampling method to collect samples of skin glucose and lactate in a group of neonates in the NICU. We were able to successfully measure glucose in 92.2% of the samples and lactate in 89.7% of the samples. Sample concentrations were found to vary depending on the feeding schedule of the baby.

Using this method, we were able to establish a strong relationship between blood glucose levels and skin glucose levels sampled at the same time, demonstrating that measurement of glucose in thermoregulatory sweat can provide a noninvasive alternative method of measuring blood glucose levels in neonates. No such correlation was found between blood lactate and skin lactate levels, suggesting that the relationship between blood and thermoregulatory sweat lactate is more complex.

The same approach was applied to monitoring healthy adults at rest. For each individual, blood glucose levels correlated well with skin glucose levels. Unlike with the neonates, this relationship varied between individuals. For a particular adult, the relationship between glucose levels in blood and thermoregulatory sweat did change slightly over time. However, once a personalized correlation line had been established, we found that levels of glucose in thermoregulatory sweat could be used to accurately predict blood glucose levels up to 28 days later with just a one-off blood measurement to correct for any change in the offset of the correlation line.

By comparison with an in vitro experimental model, we were able to establish what is happening at the skin/patch interface. Our findings illustrate the importance of considering the rate at which measurement devices remove molecules compared with the local thermoregulatory sweat rate; at high perfusion flow rates, or for large sweat sensors that consume more glucose, any results are dependent on sweat rate and sweat gland density, which can vary over time and between people. The low flow rates of our device mean that it is less dependent on sweat rate and therefore particularly suited to making measurements in thermoregulatory sweat.

We have demonstrated the potential of using this sampling method combined with online biosensors to carry out continuous measurement of glucose in thermoregulatory sweat in both healthy adults and in neonates. Continuous measurements were limited by the stability of the syringe pump at low perfusion flow rates and the high pressure caused by extension tubing. Work is ongoing to miniaturize the analysis system to create a low-volume wearable patch that is optimized for continuous measurement without interfering with clinical care.

## MATERIALS AND METHODS

### Reagents

Glucose oxidase (GOx) derived from *Aspergillus niger* and lactate oxidase (LOx) sourced from *Aerococcus viridians* were purchased from Sekisui Diagnostics, UK. Polyurethane (Texin 985) was provided by Bayer. All other reagents were acquired from Sigma-Aldrich and were of at least American Chemical Society reagent grade.

### Sampling patch

For this study, a custom-made PDMS sampling patch, featuring a teardrop-shaped sampling probe (66 Linear Microdialysis Catheter, MDialysis, Sweden), was used to collect analytes of interest from the skin, shown in Fig. 1A. For neonate monitoring, the outlet tubing length was 30 cm, and for adult monitoring, the outlet tubing length was 10 cm. The various layers of the sampling patch are shown in Fig. 1B. The 66 Linear Microdialysis Catheter, with a molecular weight cutoff of 20 kDa, includes a 30-mm semipermeable polyarylethersulphone membrane. Before applying the patch, the skin was sanitized with a surface wipe (2% chlorhexidine in 70% alcohol). The probe was secured onto a thin sheet of PDMS, shown in Fig. 1C, and connected to a 107 pump (MDialysis, Sweden), which perfused the patch with sterile T1 perfusion fluid at a flow rate of 0.5 μl/min for sample collection and 0.3 μl/min for online monitoring. The patch was secured to the skin using a hydrocolloid dressing. The outlet was either connected to miniature sample vials to collect samples for offline analysis or to our online analysis system (shown in Fig. 1D) for real-time measurements. A cross-section of the sample patch/skin interface is shown in Fig. 1E. After use, the sampling patch was cleaned with an alcohol wipe and reused if not blocked or leaking.

### Electrode fabrication

The biosensors used in this study were fabricated based on a combined needle three-electrode system described previously (*54*, *55*). Briefly, a 50-μm diameter poly(tetrafluoroethylene)-insulated platinum/iridium wire (90:10) (Advent Research Materials Ltd., UK) and a 50-μm polyester-insulated silver wire (Goodfellow Cambridge Ltd., UK) were threaded through a 27-gauge needle. Electrical connections were made using conductive silver epoxy (RS Components Ltd., UK). The needle barrel and shaft were filled with epoxy resin (CY1301 and HY1300, Robnor ResinLab Ltd., UK) to secure the wires. The needle tip was polished using sandpaper (Buehler Ltd., USA) and polished with 1-, 0.3-, and 0.05-μm alumina slurries to form two 50-μm diameter disc electrodes. The platinum disc served as the working electrode, whereas the silver disc was chloridized by immersing it in a 1 M potassium chloride solution and applying +0.24 V versus a standard Ag|AgCl reference electrode (BASi, USA) for 15 min to create a Ag|AgCl pseudo–reference electrode. Cyclic voltammetry (CV) was used to evaluate the working electrode surface. The electrode was placed in a 1.5 mM ferrocene monocarboxylate (Fc) solution, and the resulting oxidation plateau current was compared to the theoretical value (8.29 nA).

### Biosensor fabrication

Following fabrication of the combined needle microelectrodes, they were functionalized into biosensors as previously described elsewhere (*53*, *56*). Briefly, the working electrode was first coated with a poly(*m*-phenylenediamine) (mPD) layer, which serves as an exclusion layer to block potential interferents that may otherwise be oxidized at the working electrode. Electropolymerization was used to coat the mPD onto the working electrode surface by immersing the electrode in a 100 mM mPD monomer solution in 0.01 M phosphate-buffered saline (PBS) and applying a potential of +0.7 V versus a Ag|AgCl reference electrode for 20 min. After electropolymerization, the electrode was left in solution for 5 min to stabilize the mPD film and then removed and carefully rinsed with deionized water. A CV in 1.5 mM Fc was performed, and the coating was deemed successful if no oxidation peak appeared. Following successful polymerization of the mPD film, the electrode was coated with a hydrogel containing the relevant enzyme. Different hydrogel compositions were used depending on the type of biosensor (glucose, lactate). For glucose biosensors, this consisted of GOx (60 mg/ml), bovine serum albumin (BSA) (30 mg/ml), 1% (v/v) glycerol, and poly(ethylene glycol) diglycidyl ether (PEG-DE) (14.8 mg/ml) in 10 mM PBS, and for lactate biosensors, it consisted of LOx (60 mg/ml), BSA (30 mg/ml), 2% (v/v) glycerol, and PEG-DE (45.6 mg/ml) in 10 mM PBS, adapted from Vasylieva *et al.* (*57*, *58*). The electrode was dipped in the relevant hydrogel for 90 s and then placed in an oven at 55°C for 2 hours to allow cross-linking of the hydrogel matrix and immobilization of the enzyme. To extend the dynamic range of the biosensor, a diffusion-limiting polyurethane coating was applied to the biosensor surface by dip-coating. Null sensors were fabricated following the same protocol; the only difference was the composition of the hydrogel, which consisted of BSA (90 mg/ml), 1% (v/v) glycerol, and PEG-DE (14.8 mg/ml) in 10 mM PBS. After fabrication, the biosensors were stored at −20°C. The biosensors operate by the enzyme oxidizing the specific substrate to produce hydrogen peroxide. The hydrogen peroxide is then oxidized at the working electrode by applying +0.7 V versus Ag|AgCl reference electrode, and the resulting current is measured.

### Microfluidic flow cell

For all measurements presented, the biosensors were placed into a 3D-printed microfluidic flow cell, described elsewhere (*53*). The 3D-printed chip used in this study features a 350 μm–by–350 μm channel running through its center. To insert the biosensors into the microfluidic chip, they were first placed inside 3D-printed holders prior to functionalization. The biosensors were then secured in place using M3 grub screws, ensuring that the biosensor tip would be positioned in the center of the microfluidic channel when the holder was screwed into the biosensor flow cell. A schematic of the biosensors in the 3D-printed chip is shown in Fig. 1E.

### Microfluidic sample handling platform

The microfluidic sample handling platform was modified from the biosensor calibration platform described elsewhere (*59*) to allow both calibration of biosensors and analysis of skin samples. The platform consists of a LabSmith breadboard onto which are mounted four sets of syringe pumps (three 20 μl for calibration standards and T1 perfusion fluid and one 8 μl for the sample), reservoirs, and two-way valves connected together with poly(ether ether ketone) (PEEK) tubing (150 μm inner diameter, 360 μm outer diameter) as shown in fig. S1. Each syringe pump was filled with a calibration standard, T1 perfusion fluid, or a sample collected from the skin using the sampling patch. The flow streams of each met at an interconnect as a mixing junction. The biosensor calibration was carried out by adjusting the relative flow rates of the calibration standard pumps while maintaining a constant total flow rate of 0.5 μl/min; this delivered a controlled concentration of each analyte to the downstream analysis system at a flow rate that matched the perfusion flow rate. Examples of calibration traces and corresponding calibration curves obtained during the calibration phase for both glucose and lactate biosensors are shown in Fig. 2A. After calibration, skin samples could be analyzed. To do this, syringe pumps were used to alternately infuse a skin sample and T1 perfusion solution into the 3D-printed chip, creating stepwise changes in glucose and lactate levels relative to the baseline, illustrated in Fig. 2B.

### Offline sample analysis

The discontinuous skin samples were sequentially infused through a 3D-printed chip containing a null sensor, a glucose sensor, and a lactate sensor. The biosensors were controlled using custom high-performance potentiostats connected to a PowerLab 8/35 and controlled by LabChart Pro (ADInstruments, Sydney, Australia). The potentiostats were used to hold the working electrodes at +0.7 V versus a Ag|AgCl reference electrode and to record biosensor output currents. All signals were recorded with a sampling frequency of 200 Hz and a 10-Hz low-pass filter. Sensor currents for glucose and lactate were baseline-corrected and converted into concentrations using a calibration curve obtained during the analysis. For each sample, the average concentration during the plateau phase was calculated. These values were visualized as a histogram with multiple bars, each representing a separate skin sample collected at different time points. The time is corrected to account for transit delay and therefore reflects the actual sampling intervals, aligned to the original sampling time rather than the time the samples reached the collection vial. For longer collection periods with larger sample volumes, each sample was analyzed multiple times and the values of repeat plateaus averaged together. For shorter 20-min collections, the sample volume was limited to 10 μl. Therefore, only a single measurement was performed for each sample.

### Online analysis

During wireless continuous monitoring, the sampling patch was perfused with T1 perfusion fluid at a flow rate of 0.3 μl/min via a 107 pump (MDialysis, Sweden) and inlet extension tubing. The outlet of the sampling patch was connected to the 3D-printed microfluidic chip housing a glucose and lactate biosensor within its channel. Wireless potentiostats, which have been described elsewhere (*52*, *56*), were used to apply a +0.7 V potential versus a Ag|AgCl reference electrode to the biosensor working electrode. Each potentiostat, designed in-house and implemented on a custom two-layer battery-powered (3.7 V rechargeable lithium-ion battery, 1.8 A·hour, BAK) printed circuit board (PCB), featured two amperometric channels with selectable gain settings (1, 0.1, and 0.2 nA/V). Currents were sampled at 10 Hz and transmitted in real time via Bluetooth to a Samsung tablet (SAMSUNG Galaxy Tab A 9.7”), where data were visualized using a custom Android application. To monitor glucose and lactate simultaneously, one potentiostat housed in a custom 3D-printed enclosure was used with the pump secured to the side. Biosensor calibration was performed both before and after continuous monitoring to assess sensor performance. Glucose and lactate currents were converted to concentration by applying the respective calibration curves to baseline-corrected current signals. A time correction was applied to account for transit delay, enabling near real-time visualization of analyte levels.

### Neonate protocol

The study protocol was reviewed and approved by UK Health Research Authority in accordance with the UK Policy framework for health and social care and Imperial College’s Joint Research Compliance Office (REC reference no: 21/PR/0778). Research nurses approached parents of newborn babies fitting the study criteria to explain the study, to give them the patient information sheet to review, and to answer any questions. If the parents decided to take part, written informed consent was obtained from the parents of all recruited babies prior to any research procedures being undertaken. Inclusion criteria were small for GA preterm babies born between 25 to 34 weeks of GA requiring admission to the neonatal unit and babies born of mothers with gestational diabetes mellitus requiring admission to the neonatal unit for management. Exclusion criteria were babies with major congenital abnormalities including life-limiting chromosomal and genetic disorders, babies who are unstable and not deemed suitable for additional probe placements by the attending clinical team, and moribund babies or those undergoing palliative care. Table S1 gives the GA and postnatal age for all babies monitored. For each baby, the GA refers to the duration of pregnancy in weeks and days, whereas the postnatal age refers to the chronological age of the baby in days since birth. Corrected GA is the GA at birth corrected by adding the postnatal age expressed in weeks and days. All equipment was wiped with ethanol prior to entering the NICU. The sampling patch was preflushed with T1 perfusion fluid using a 107 pump (MDialysis, Sweden) via an inlet extension before placement on the infant’s abdomen or back, as shown in Fig. 1 (F and G). Analysis was either conducted online with a perfusion flow rate of 0.3 μl/min or samples were collected with a perfusion flow rate of 0.5 μl/min for offline analysis. For offline analysis, 15 min after patch placement, a collection vial was connected to the outlet tubing to allow time for the skin sample to reach the tubing outlet. Vials were replaced at fixed intervals, and all collected samples were stored at −20°C until analysis. For real-time online analysis, the outlet tubing was instead connected to a 3D-printed microfluidic chip prior to patch placement on the infant’s skin. Blood samples were taken as part of routine clinical care, and analysis of glucose and lactate levels was carried out using an arterial blood gas analyzer. Where possible, blood measurements were mostly taken immediately before or after a feed where levels would have been more stable.

### Adult protocol

This study was conducted in accordance with the Declaration of Helsinki and was approved by the Imperial College Research Ethics Committee (19IC4999). Skin was wiped with alcohol to remove bacteria from the skin. Analysis was either conducted online or samples were collected for offline analysis. For offline sample collection, T1 perfusion liquid was preflushed directly from a 107 pump at 0.5 μl/min through the sampling patch, which was then placed on the ventral forearm of adult volunteers. Ten minutes after patch placement, a collection vial was connected to the outlet tubing to allow time for the skin sample to reach the tubing outlet. The perfusate was collected in vials every 20 min. All samples were stored at −20°C until analysis. Blood glucose levels were measured at the start of each collection period using a True Metrix GO glucometer (Trividia Health, UK). For online monitoring, the sampling patch was perfused at 0.3 μl/min. It was positioned on the forearm before lunch, and the outlet tubing was connected to a 3D-printed microfluidic chip. Once the glucose signal had stabilized, participants were asked to consume chocolate or a sugar-rich beverage and lunch, to induce an increase in blood glucose levels. Monitoring continued until both blood and skin glucose levels returned to baseline. At the end of the experiment, the sampling patch was removed, flushed with distilled water, and disinfected with an alcohol wipe. Blood glucose was measured every 10 to 15 min.

### Visualizing sweat gland density

Bromophenol blue dye was used to visualize sweat gland density on the ventral forearm of healthy adults as described elsewhere (*32*, *60*). Briefly, bromophenol blue was dissolved in acetone in a 5% (w/v) solution. This was mixed with silicone in a 1:1 volumetric ratio and stirred until the acetone had evaporated. A thin layer of the orange paste was applied to the ventral forearm and left for a few minutes until purple dots began to be visible where thermoregulatory sweat was produced. Photos were captured using a handheld digital microscope (Dino-Lite, Taiwan).
