# Supplementary material for: Noninvasive measurement of glucose and lactate in thermoregulatory sweat in neonates and adults
Source: Sci Adv. 2026 Jul 15;12(29):eaec4166. doi: 10.1126/sciadv.aec4166 (PMC13371885; doi:10.1126/sciadv.aec4166)
Supplement: Supplementary file 1 — Supplementary Text Figs. S1 to S10 Tables S1 and S2 Legend for movie S1 [file sciadv.aec4166_sm.pdf]

Science Advances
AAAS

Supplementary Materials for

Noninvasive measurement of glucose and lactate in thermoregulatory sweat in neonates and adults

Xinyue Liu *et al.*

Corresponding author: Martyn G. Boutelle, m.boutelle@imperial.ac.uk

Sci. Adv. **12**, eaec4166 (2026)
DOI: 10.1126/sciadv.aec4166

The PDF file includes:

Supplementary Text
Figs. S1 to S10
Tables S1 and S2
Legend for movie S1

Other Supplementary Material for this manuscript includes the following:

Movie S1

Sample analysis

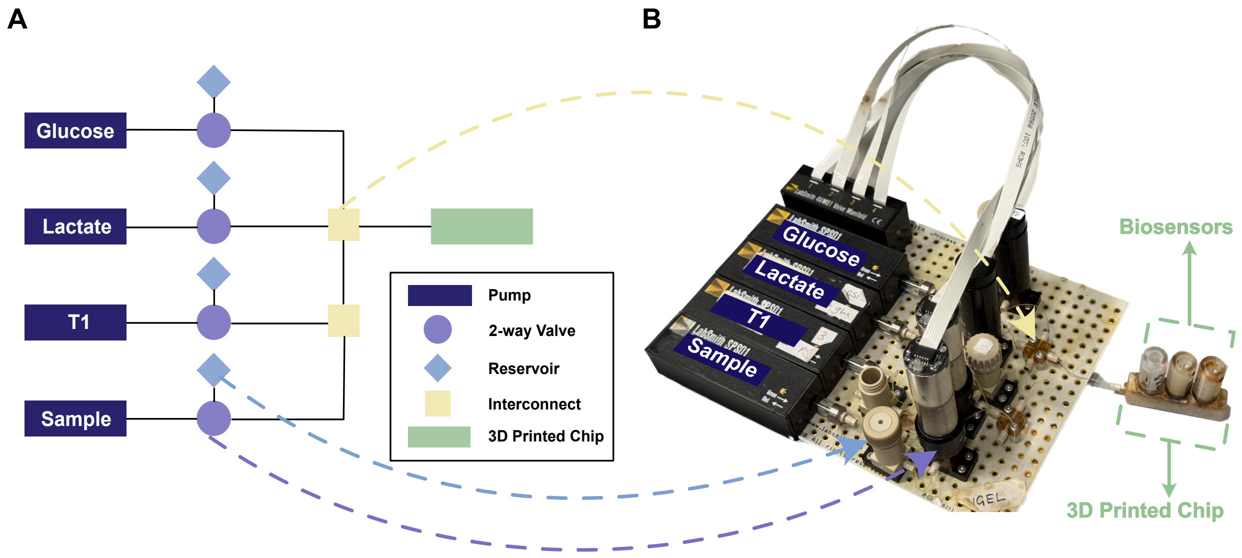

Figure SI1A. Schematic of microfluidic sample handling platform connected to 3D printed microfluidic chip containing biosensors for glucose and lactate. It consists of four programmable syringe pumps and four 2-way valves each connected to a reservoir containing either glucose standard, lactate standard, T1 or an unknown sample. The glucose and lactate streams connect at an interconnect and their relative flow rates are varied to control the overall concentration of glucose and lactate pushed into the 3D printed chip and past the glucose and lactate biosensors at a constant flow rate. The T1 and sample streams are used to alternately flow T1 solution and 10 µl aliquots of a sample into the 3D printed chip and past the biosensors. A minimum sample volume of 10 µl is required for this analysis. B. Photo of microfluidic sample handling platform. Arrows from the schematic indicate the valve, reservoir and interconnect.

Patient information

Baby Number	Gestational Age (Week+Day)	Postnatal Age (Day)	Corrected GA (Week+Day)
Baby 1	27+5	41	33+4
Baby 2	25+3	95	39+0
Baby 3	23+5	61	32+3
Baby 4	26+6	37	32+1
Baby 5	26+6	37	32+1
Baby 6	30+1	36	35+2
Baby 7	38+2	18	40+6
Baby 8	24+0	70	34+0
Baby 9	24+0	69	33+6
Baby 10	26+3	52	33+6
Baby 11	27+1	31	31+4
Baby 12	28+6	19	31+4
Baby 13	30+0	11	31+4
Baby 14	32+1	2	32+3
Baby 15	28+0	7	29+0
Baby 16	32+5	3	33+1
Baby 17	33+2	2	33+4
Baby 18	23+4	27	27+3
Baby 19	27+0	19	29+5
Baby 20	23+3	21	26+3
Baby 21	30+0	7	31+0
Baby 22	30+0	7	31+0
Baby 23	33+2	6	34+1
Baby 24	33+2	6	34+1
Baby 25	26+0	54	33+5
Baby 26	26+3	8	27+4
Baby 27	31+3	24	34+6
Baby 28	32+4	11	34+1

Table SI1. Gestational age (GA), postnatal age and corrected GA for each baby monitored.

Low time resolution neonate measurements

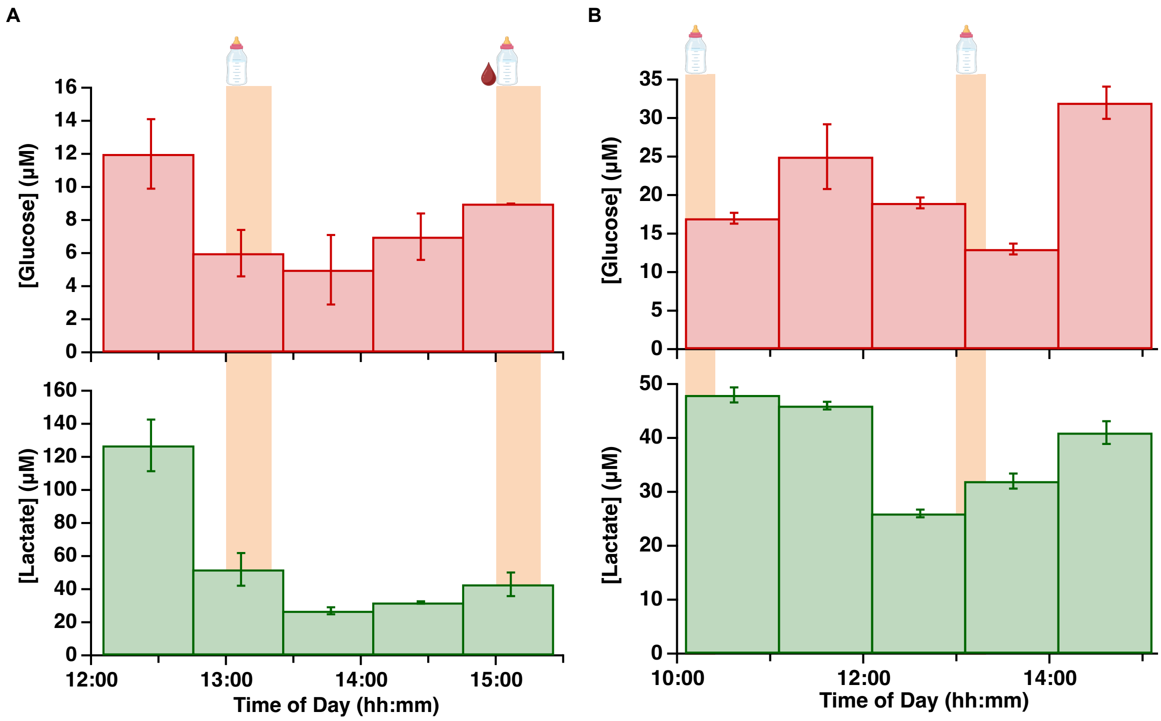

Figure SI2. Examples of glucose (red) and lactate (green) levels in samples collected on the skin of two babies. Each bar represents the time that sample was collected over. Each sample is 40 min long for A and 60 min long for B. Bars and error bars represent the mean and standard deviation of the measurement for each sample. Samples have been shifted to account for the delay between the membrane and the sample vial (15 min). Nasogastric feeds typically lasted 20 min and the start is indicated by an image of a milk bottle. The duration is indicated by the orange bars under the bar graphs. An image of a blood droplet indicates when a blood measurement was made. A. In this example the baby was fed every 2 hours and the baby was 26+3 weeks gestational age and 52 days postnatal age. B. In this example the baby was 24+0 weeks gestational age and 83 days postnatal age and was fed on a 3-hourly schedule.

Example sweat gland density on adult ventral forearm

Bromophenol blue dye was used to visualise the sweat gland density of three adults tested in figure 4. The method is described in the methods and materials section. Briefly a silicone paste containing bromophenol blue (orange dye) was applied to the ventral forearm while the adult was at rest. The dye turned purple as sweat was produced, enabling visualisation of the sweat glands. Photos were taken using a handheld microscope so that the sweat glands could be counted and the sweat gland density calculated. Each photo covered an area of between 0.2-1 cm^2. An example photo from adult 4 is shown below in figure SI3.

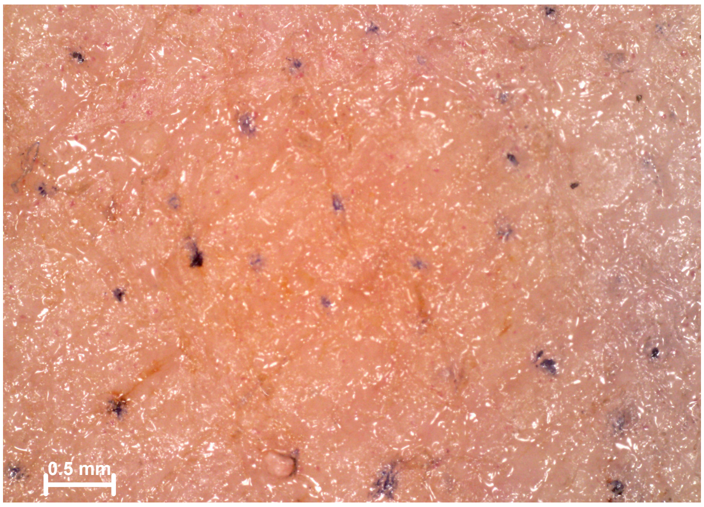

Figure SI3. Example sweat gland density on adult 4 ventral forearm at rest. Sweat glands are shown in purple. To visualise the sweat glands we followed the protocol described in the methods and materials section.

A summary of the sweat gland density for 3 adults shown in figure 4 is described below in table SI2. The correlation lines of sweat and blood measurements for adults 1 and 4 are considerably steeper than for adult 6, which is consistent with the higher sweat gland density for adults 1 and 4. The difference in sweat gland density, however, does not explain the difference in correlation slope between adults 1 and 4, suggesting that the relationship is not simple.

Adult Number	Sweat Gland Density ± SD	Number of Photos
Adult 1	107.6 ± 16.8	7
Adult 4	177.1 ± 40.3	7
Adult 6	44.1 ± 6.0	34

Table SI2. Mean sweat gland density ± standard deviation for three adults shown in figure 4. Sweat glands were visualised using bromophenol blue dye. The number of sweat glands were counted and the sweat gland density calculated for each photo. The mean and standard deviation across all the photos was calculated.

An example showing real-time production of thermoregulatory sweat visualised using bromophenol blue is shown in the attached video (Sweat gland video.mp4). In this case the

dye is applied to the palm of the hand for visualisation purposes as there is a higher sweat gland density in this area.

Offline skin and blood glucose measurements from adults on identical scales

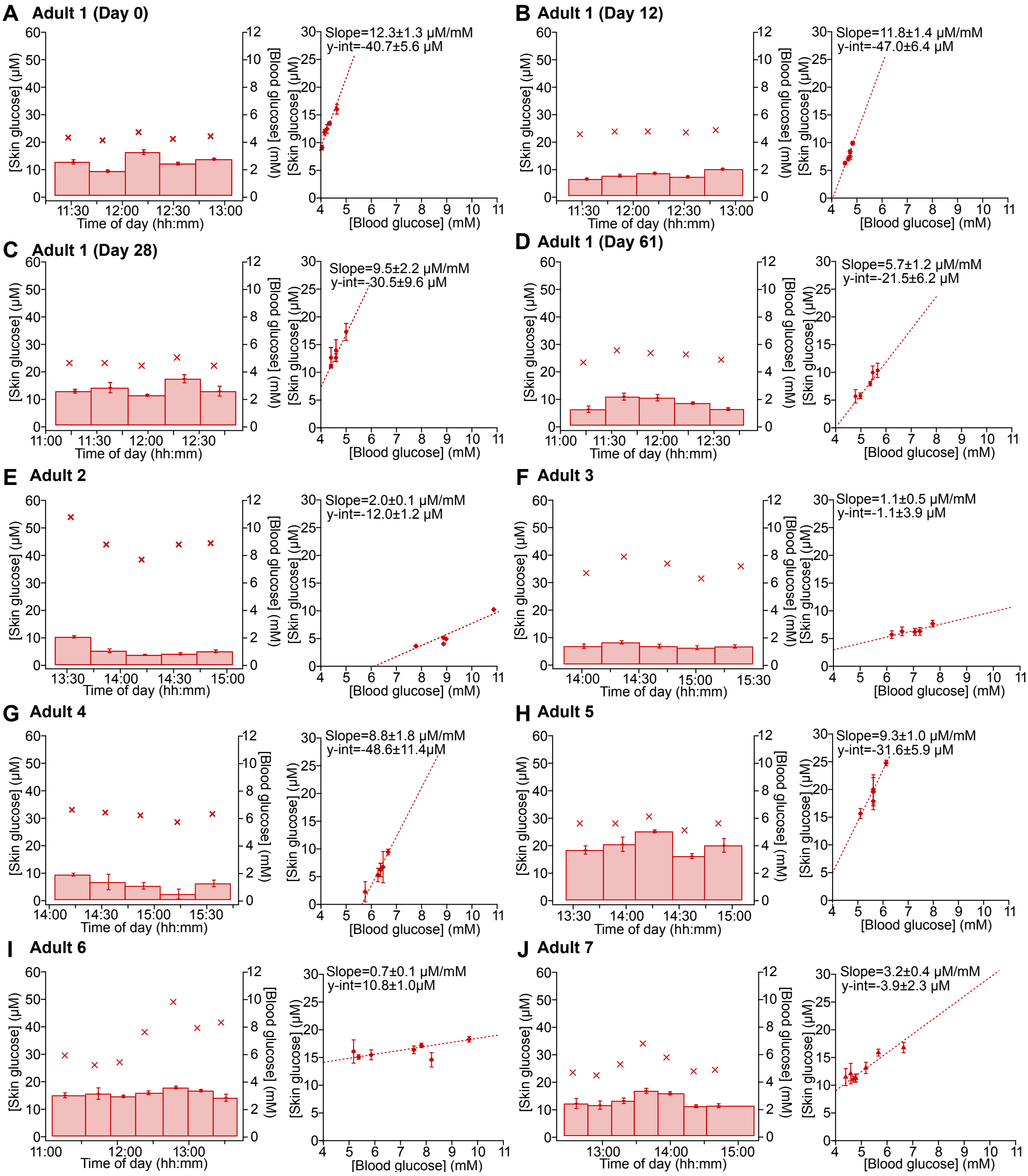

Figure SI4. Glucose levels measured from skin samples of seven adults. Real-time measurement traces for all participants are presented using identical axis scales, and correlation plots are likewise shown using consistent scales across adults. A-D. Examples of glucose levels in samples collected on the skin of one adult at rest over four separate occasions (bars) and concomitant blood glucose levels (crosses). Scatter graph shows the correlation between skin sample glucose concentration and blood glucose concentration at each time point: at A. day 0, B. day 12, C. day 28 and D. day 61. E-J. Examples of glucose levels in samples collected on the skin of a further six adults. In each case, each bar represents the time that sample was collected over. Each sample is 20 min long. Bars and error bars represent the mean and standard deviation of the plateau measurement for each sample. Samples have been shifted to account for the delay between the patch and the sample vial (7

min). Crosses indicate the glucose concentration in one-off finger-prick blood tests, taken roughly midway through each skin sample with a blood glucose analyser. This is plotted against the sample concentration on the adult's skin at the same time as the blood sample is taken. The points are fitted with a weighted linear regression for each adult. The regression coefficients ± standard deviation of the slope and y-intercept are given. The correlation lines for adult 1 have slopes of 12.3 ± 1.3 μM/mM (R^2 = 0.91) for day 0, 11.8 ± 1.4 μM/mM (R^2 = 0.84) for day 12, 9.5 ± 2.2 μM/mM (R^2 = 0.80) for day 28 and 6.2 ± 1.2 μM/mM (R^2 = 0.82) for day 61. The slopes for adults 2-7 are 2.0 ± 0.1 μM/mM (R^2 = 0.82), 1.1 ± 0.5 μM/mM (R^2 = 0.86), 8.8 ± 1.8 μM/mM (R^2 = 0.93), 9.3 ± 1.0 μM/mM (R^2 = 0.87), 0.7 ± 0.1 μM/mM (R^2 = 0.84), 3.2 ± 0.4 μM/mM (R^2 = 0.84), respectively.

Overall adult measurements

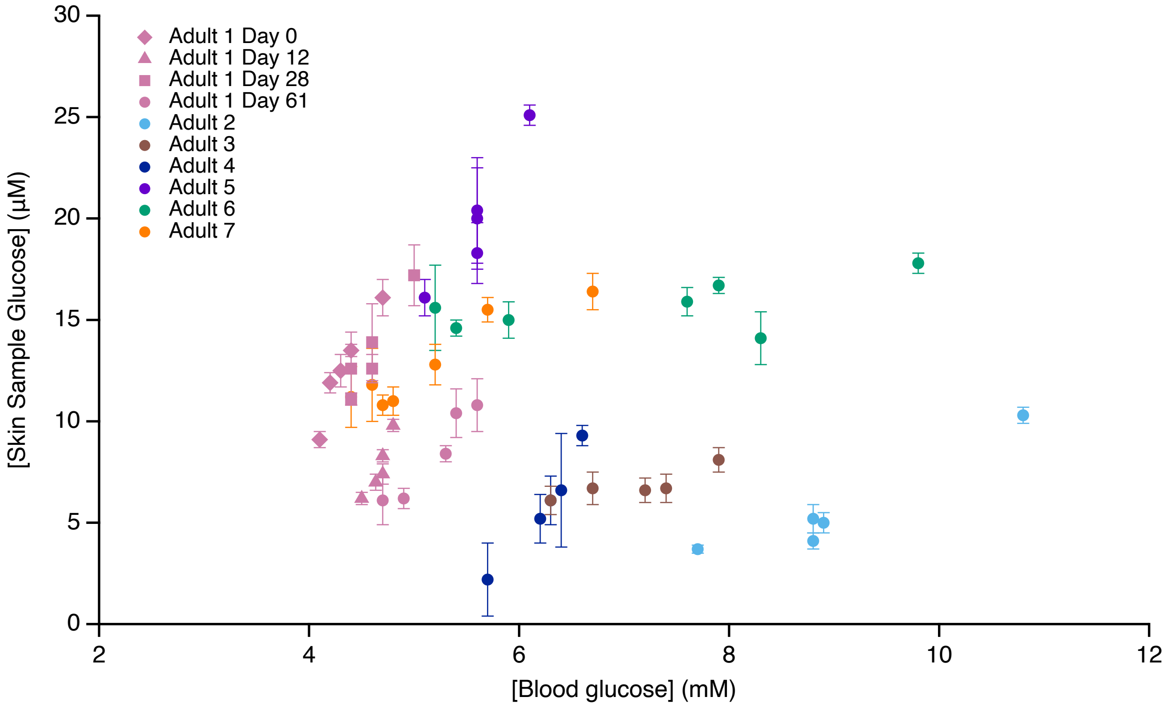

Figure SI5. Overall plot showing correlation between skin sample glucose concentration and blood glucose concentration for all adults. Markers and error bars represent the mean and standard deviation of the measurement. Different colours correspond to different adults.

Box plots of glucose concentrations in blood and skin samples from neonates and adults

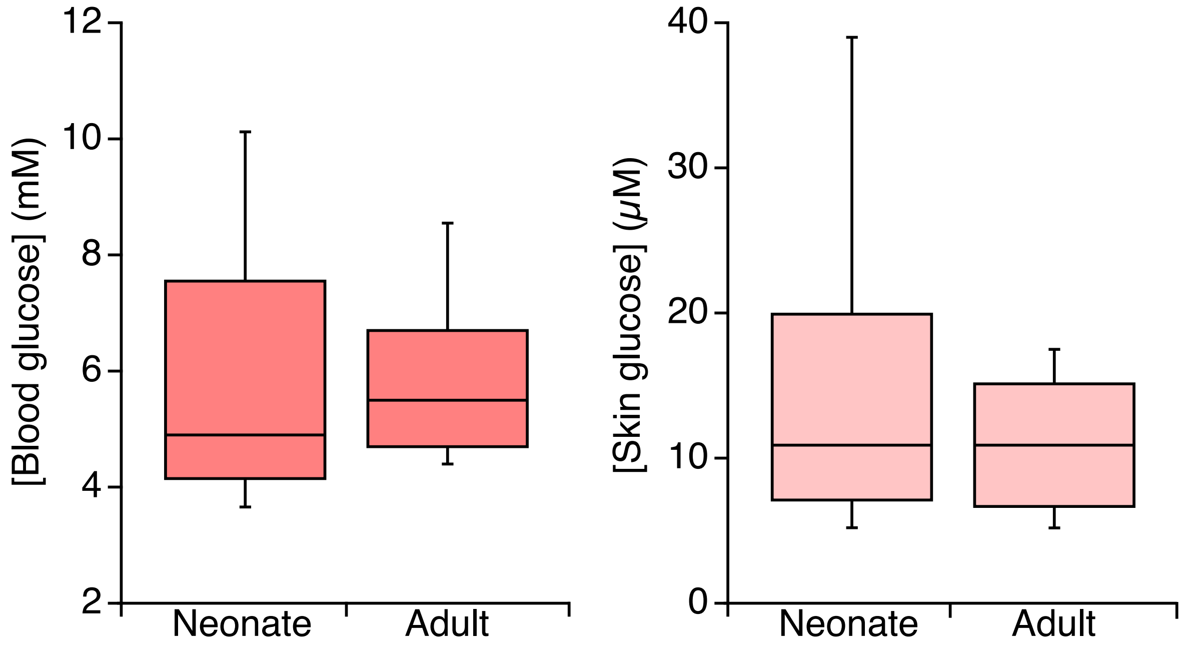

Figure SI6. Box plots comparing the blood glucose concentration (left, red) and skin sample glucose concentration (right, pink) between neonates and adults monitored. Left red box plot shows the concentration of blood glucose (n=17 samples) for 15 babies. Right red box plot shows the concentration of blood glucose (n=54 samples) for all samples measured from 7 adult participants. Left pink box plot shows the concentration of glucose (n=224 samples) in offline skin samples analysed from 28 babies. In total, 19 samples were excluded for glucose and 25 samples were excluded for lactate as their levels were below the limit of detection of the biosensor used for that experiment and therefore indistinguishable from zero. Right pink box plot shows the concentration of glucose (n=54 samples) in all skin samples analysed from 7 adults. Boxes represent median and interquartile range and whiskers represent 10th and 90th percentiles.

Long-term blood glucose prediction

Figure SI7 shows that over longer time periods applying a previous correlation between sweat and blood glucose levels to predict blood levels from sweat measurements is less effective; the predicted blood glucose levels on day 61 calculated using the relationship between sweat and blood glucose levels at day 0 are considerably lower than the actual blood glucose levels, and the slope of the predicted blood glucose level to actual blood glucose level is 0.5 ± 0.08. Using a one-point blood measurement to correct for the change in offset improves the predicted values and although the points are further away from the actual values than at days 12 or 28, they are within the margin of error of the blood glucose meter.

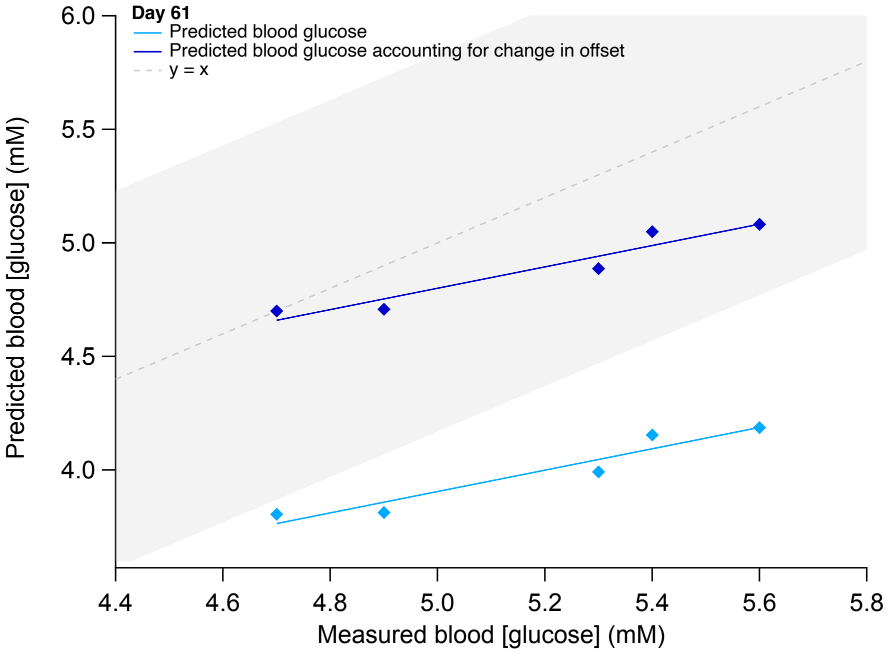

Figure SI7. Correlation between predicted blood glucose levels on day 61 against measured blood glucose concentration (light blue). Predicted blood glucose concentrations are calculated from skin sample glucose levels using the regression line between skin sample glucose and blood glucose on day 0. Using a one-point blood measurement on day 61 the offset can be accounted for (dark blue). Markers and error bars represent the mean and standard deviation of each measurement. Points are fitted with a weighted regression line. The grey dotted line represents where predicted blood glucose levels and measured blood glucose levels are equal. The shaded grey area represents the margin of error in the accuracy of the blood glucose meter (±0.83 mM for glucose levels <5.55 mM).

On-skin extrapolation to zero flow

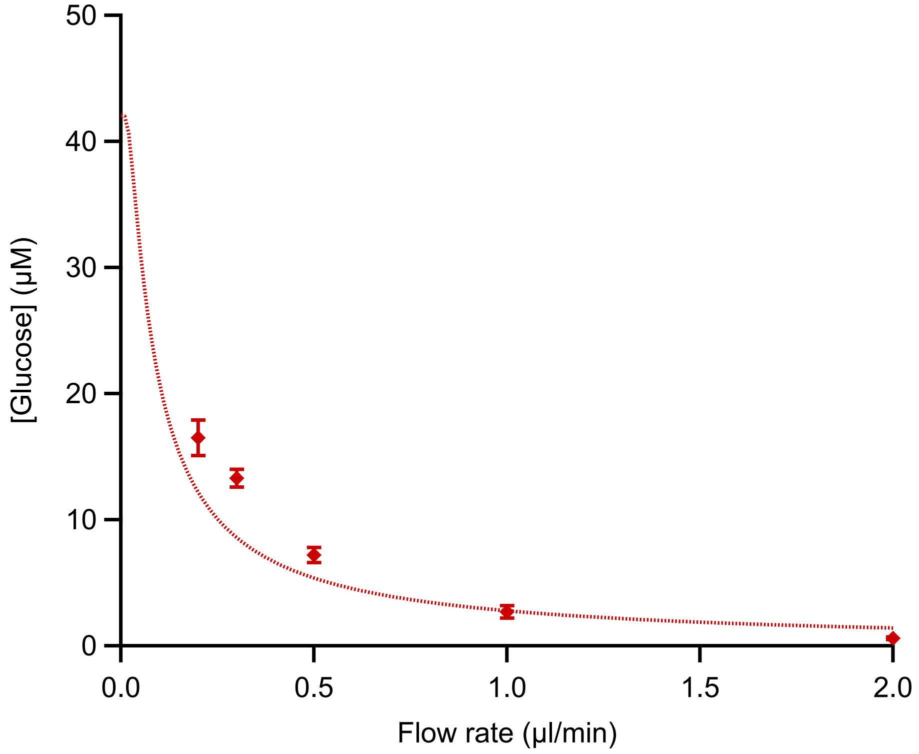

Figure SI8. Variation in glucose concentration in sample with flow rate from 0.2 µl/min to 2 µl/min when sampling patch is placed on the forearm of a healthy adult. The points are fitted with the extrapolation to zero flow rate equation, $C_{out} = C_{ext} - C_{ext}\, e^{(-k0A/F)}$, where C_{out} is the concentration of glucose in the sample, C_{ext} is the actual concentration of glucose on the skin, k_0 is the average mass transfer coefficient, A is the area of the probe, and F is the flow rate. Markers and error bars represent the mean and standard deviation of each measurement once it stabilised. For each measurement the mean current was converted to a glucose concentration using a calibration carried out at the same flow rate.

Correlation between target flow rate and actual flow rate

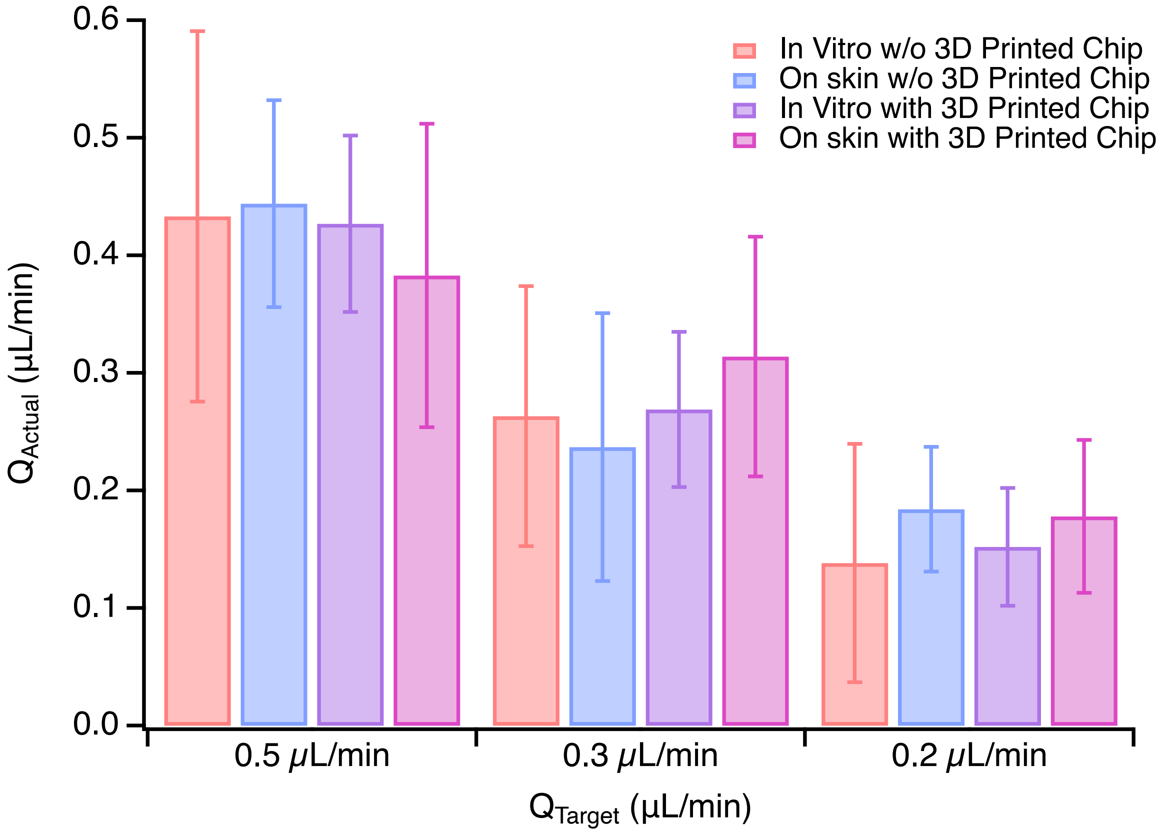

Figure SI9. Variation in measured flow rate (Q_{Actual}) for four different conditions for 0.5, 0.3 and 0.2 µl/min target flow rates (Q_{Target}). Bars and error bars represent the mean and standard deviation for each measurement. For all conditions, the outlet of the sampling patch was connected to the inlet of a flow meter (LG16-0150D, Sensirion AG, Switzerland), and the flow rate was measured in real time using the USB RS485 Sensor Viewer software. The red bars show when the outlet of the flow meter was not connected to anything (left open to the atmosphere) and the patch was placed on a petri dish. The blue bars show when the outlet of the flow meter was not connected to anything (left open to the atmosphere) and the patch was placed on the arm of a healthy adult. The purple bars show when the outlet of the flow meter was connected to the inlet of the 3D printed microfluidic chip and the patch was placed on a petri dish. The green bars show when the outlet of the flow meter was connected to the inlet of the 3D printed microfluidic chip and the patch was placed on the arm of a healthy adult. The large error represents fluctuations in the flow rate due to the syringe pump.

Lower temporal resolution online adult monitoring

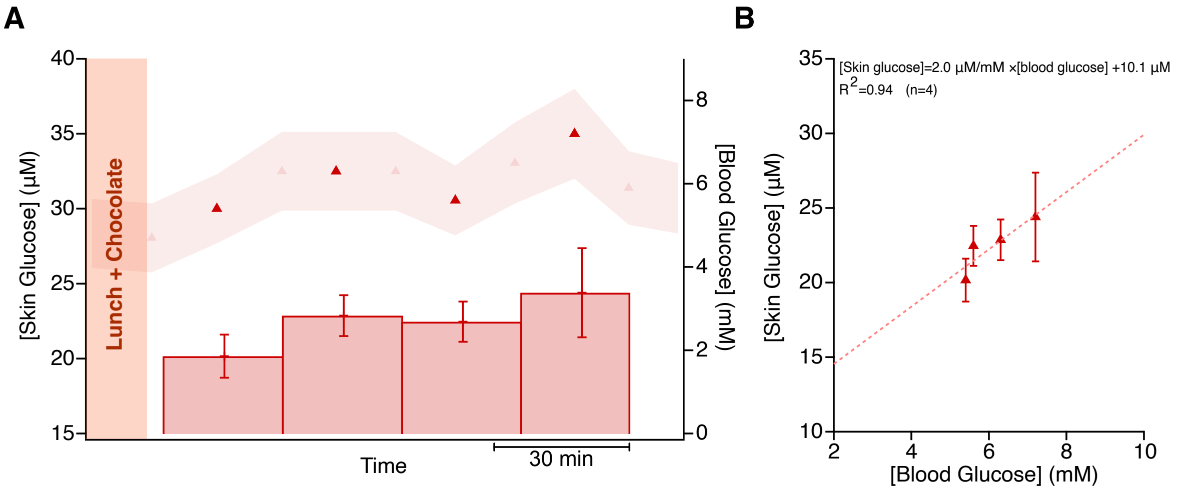

Figure SI10. Averaging of online adult skin glucose monitoring into 20-minute time windows to mimic offline sample analysis. A. Skin glucose concentrations were averaged into 20-minute windows centred on each blood sampling time point (±10 minutes). Bars and error bars indicate the mean and standard deviation of the skin glucose concentration within each time window. An average was taken corresponding to every other blood measurement so as not to overlap the averages. Corresponding blood glucose measurements are shown as dark red triangles. B. Scatter graph showing the correlation between skin glucose concentration and blood glucose concentration. Markers and error bars represent the mean and standard deviation of the measurement for each time window. Points are fitted with a weighted regression line. The correlation line has a slope of 2.0 ± 1.5 µM/mM (R^2 = 0.94).

Visualizing sweat glands

Video S1. Human sweat glands are visualised producing thermoregulatory sweat under a microscope using bromophenol blue dye. The dye is orange upon application to the skin and turns purple when sweat is produced. The video is sped up by 10 times.
